# Thymosin β4 reverses phenotypic polarization of glial cells and cognitive impairment via negative regulation of NF-κB signaling axis in APP/PS1 mice

**DOI:** 10.1186/s12974-021-02166-3

**Published:** 2021-06-28

**Authors:** Meng Wang, Li-Rong Feng, Zi-Long Li, Kai-Ge Ma, Ke-Wei Chang, Xin-Lin Chen, Peng-Bo Yang, Sheng-Feng Ji, Yan-Bing Ma, Hua Han, John Bosco Ruganzua, Wei-Na Yang, Yi-Hua Qian

**Affiliations:** 1 Department of Human Anatomy and Histology-Embryology, School of Basic Medical Sciences, Xi’an Jiaotong University Health Science Center, 76 Yanta West Road, Xi’an, 710061 Shaanxi China; 2 Institute of Neuroscience, School of Basic Medical Sciences, Xi’an Jiaotong University Health Science Center, Xi’an, China; 3 Key Laboratory of Environment and Genes Related to Diseases (Xi’an Jiaotong University), Ministry of Education of China, Xi’an Jiaotong University Health Science Center, 76 Yanta West Road, Xi’an, 710061 Shaanxi China; 4 Institute of Neurobiology, Xi’an Jiaotong University Health Science Center, Xi’an, China

**Keywords:** Thymosin β4, Alzheimer’s disease, Cognition and emotion, Phenotypic polarization of glial cells and neuroinflammation, NF-κB signaling pathway

## Abstract

**Background:**

Thymosin β4 (Tβ4) is the most abundant member of the β-thymosins and plays an important role in the control of actin polymerization in eukaryotic cells. While its effects in multiple organs and diseases are being widely investigated, the safety profile has been established in animals and humans, currently, little is known about its influence on Alzheimer’s disease (AD) and the possible mechanisms. Thus, we aimed to evaluate the effects and mechanisms of Tβ4 on glial polarization and cognitive performance in APP/PS1 transgenic mice.

**Methods:**

Behavior tests were conducted to assess the learning and memory, anxiety and depression in APP/PS1 mice. Thioflavin S staining, Nissl staining, immunohistochemistry/immunofluorescence, ELISA, qRT-PCR, and immunoblotting were performed to explore Aβ accumulation, phenotypic polarization of glial cells, neuronal loss and function, and TLR4/NF-κB axis in APP/PS1 mice.

**Results:**

We demonstrated that Tβ4 protein level elevated in all APP/PS1 mice. Over-expression of Tβ4 alone alleviated AD-like phenotypes of APP/PS1 mice, showed less brain Aβ accumulation and more Insulin-degrading enzyme (IDE), reversed phenotypic polarization of microglia and astrocyte to a healthy state, improved neuronal function and cognitive behavior performance, and accidentally displayed antidepressant-like effect. Besides, Tβ4 could downregulate both TLR4/MyD88/NF-κB p65 and p52-dependent inflammatory pathways in the APP/PS1 mice. While combination drug of TLR4 antagonist TAK242 or NF-κB p65 inhibitor PDTC exerted no further effects.

**Conclusions:**

These results suggest that Tβ4 may exert its function by regulating both classical and non-canonical NF-κB signaling and is restoring its function as a potential therapeutic target against AD.

**Supplementary Information:**

The online version contains supplementary material available at 10.1186/s12974-021-02166-3.

## Background

Alzheimer’s disease (AD) is the leading cause of dementia and is characterized by progressive learning and memory decline, often accompanied by mental disorders, such as emotional apathy, anxiety, and depression [1, 2]. Neuroinflammation has garnered substantial public attention because AD pathogenesis includes strong interactions with immunological mechanisms in the brain [3]. The imbalance of phenotypic polarization of glial cells is the central event of diffuse inflammation in AD brain [4].

Studies have found that in the normal process of neural circuit formation, M2- phenotype of microglia mediated the pruning of synapses through phagocytosis, revealing the role of microglia in promoting the remodeling and maturation of synaptic circuits, showing an anti-inflammatory effect [5, 6]. While in the immune microenvironment of AD, the shift of the M2-phenotype to the pro-inflammatory M1-phenotype is observed, resulting in a pro-inflammatory effect [7, 8]. The activated M1 microglia can further promote astrocyte shifting to the A1 phenotype [9], which loses the normal function and produce complement, releases neurotoxic factors [10]. The inflammatory mediators released by the reactive cells may in turn stimulate amyloid β (Aβ) production and aggregation [7]. They thus synergistically accelerate abnormal CNS function, culminating in enhanced cognitive deficits [11]. Therefore, deciphering the interplay between phenotypic polarization and AD may help designing anti-inflammatory target to control the action of polymorphic glial cells in triggering the disease.

Thymosin β4 (Tβ4) is a polypeptide composed of 43 amino acids [12], a constituent part of cytoplasm and cytoskeleton that is also distributed in the brain [13]. The gene ontology hinted that the peptide could be endowed with functions as diverse as anti-inflammation and cell motility, promoting axonal growth in dendritic spines, as well as synaptogenesis or plastic changes [14, 15]. Researchers proposed that the treatment potential of Tβ4 includes neurodegenerative diseases, stroke, spinal cord injury, chronic pain, and psychiatric disorders such as anxiety, depression, and schizophrenia [16]. Besides, the safety profile of Tβ4 has been established in animals and humans [17]. Altogether, these studies demonstrate that Tβ4 is a prospective candidate for AD. Unfortunately, direct animal investigations of whether and how Tβ4 impacts on behaviors that are impaired in psychiatric and neurodegenerative diseases, like AD, are lacking [18]. Here, we established a steady gene delivery of Tβ4 targeting to neuronal cells to investigate the interplay between Tβ4 and neuroinflammation in AD treatment.

## Materials and methods

### Animals

Eight-month-old APP695/PS1-dE9 (APP/PS1) double transgenic mice, and their age- and gender-matched wild-type (WT) littermates were employed in this experiment. All cohorts consisted of 50% female mice. Male APP/PS1 mice (8 weeks) and wild type female mice with the same genetic background (8 weeks) were originally obtained from the Model Animal Research Center of Nanjing University (Nanjing, China) and then kindly provided by associate professor Wei-Na Yang in 2018. All mice were bred under standard conditions (12-h light-dark cycle, room temperature 23 ± 1 °C, humidity 50 ± 5%, access to food and water ad libitum) before testing. The experiments were carried out in compliance with The Guidelines for Animal Care and Use of China, and the experimental protocols were approved by Xi’an Jiaotong University Institutional Animal Care and Use Committee. Animal suffering was minimized during the experiment.

### Lentivirus and drugs

The lentivirus including LV-CON and LV-Tβ4-overexpression (mouse Tβ4 gene, NCBI ID: NM_012209) were provided by Gene chem Co., Ltd. (Shanghai). The titer for LV-CON was 1.5 × 10^8^ TU/mL, and the titer for LV-Tβ4-overexpressing was 2.0 × 10^8^ TU/mL. The TLR4 antagonist TAK242 (Med Chem Express, 243984-11-4) and NF-κB p65 inhibitor PDTC (Med Chem Express, 5108-96-3) were dissolved in the clarified mixture of 10% DMSO (Sigma-Aldrich, C6164) and 90% corn oil (Med Chem Express, 8001-30-7). The chemical compounds under study were endotoxin-free.

### Grouping and treatment

Animals were randomly assigned to 5 groups: WT (LV-CON plus intraperitoneal (i.p.) injection of DMSO and corn oil), APP/PS1 (LV-CON plus i.p. injection of DMSO and corn oil), APP/PS1 + Tβ4 (LV-Tβ4 plus i.p. injection of DMSO and corn oil), APP/PS1 + Tβ4 + TAK242 (LV-Tβ4 plus i.p. injection of TAK242, 3 mg/kg/day) [19, 20], and APP/PS1+Tβ4 + PDTC (Tβ4-LV plus i.p. injection of PDTC, 50 mg/kg/day) [21, 22].

**Fig. 1:**
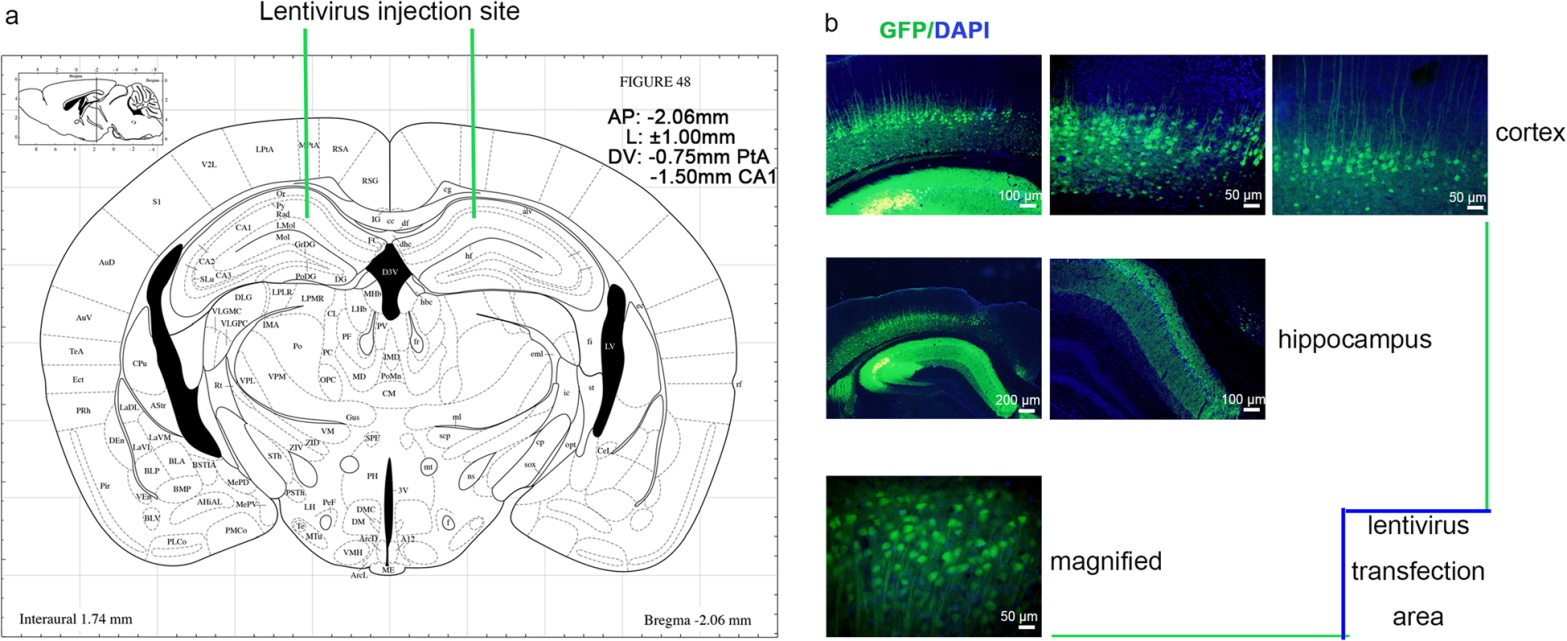
Bilateral brain injections of lentivirus. **a** Coronal diagram showing the field of the microinjection located in the subregions (PtA and CA1). **b** The field of lentivirus transfection field

For lentivirus brain stereotactic injection, mice were anaesthetized by isoflurane inhalation and transferred to a brain stereotactic apparatus (RWD Life science Co, Ltd; Shenzhen, China). The same quantity of lentivirus was injected into the bilateral hippocampus CA1 region (AP = − 2.06 mm, L = 1.0 mm, and DV = − 1.50 mm) and cortex PtA (parietal association cortex) region (AP = − 2.06 mm, L = 1.0 mm, and DV = − 0.75 mm) by a stainless glass electrode welding in a microsyringe at a rate of 0.3 μL/min according to the coordinates of mouse cerebral atlas [23, 24] (Fig. 1a). The needle was left in place for another 5 min and then slowly withdrawn. The needle placement for the animal was histologically verified. Mice were subjected to intraperitoneal injection of the two chemicals or corresponding volume of an appropriate vehicle for 5 days before behavioral tests until the last day of the behavioral tests, the procedure was repeated daily. The intraperitoneal injection for each mouse was 8 h before its behavioral experiments when the behavioral experiments began according to the pharmacokinetics of chemicals [25, 26].

All behavioral tests were performed during the light cycle. The cortex and hippocampus of mice were collected after behavioral experiments for protein and mRNA detection.

### Behavioral assays

#### Open field test

Before the behavioral assays, mice were handled for 5 min each for 3 days to habituate the animals to the experimenter and reduce anxiety. The open-field test (OFT) was used to assess autonomous exploration behaviors in a novel environment [27]. The field was 30-cm-high, open-square grey area (40 × 40 cm), with clear Plexiglas walls, in a quiet, dimly lit room. The experiment was being tracked by the SMART tracking program (San Diego Instruments, San Diego, CA). The OFT started with a habituation trial of 10 min during which the animals were individually introduced in the center of the empty arena and were free to explore the new environment. The total distance and average speed were record and analyzed. The surface of the arena was cleaned with 70% alcohol between tests.

#### Cognitive behavioral tests

The novel object recognition test (NORT) is to assess short-term declarative memory [28]. Mice were allowed to 10 min acquisition period with two identical objects, then followed by a 1-h retention period and a 10-min trial phase, which involved replacing one of the objects with a novel object. During the trial phase the orofacial exploration (whisking or sniffing) time of the familiar and the novel object spent by mice were measured by a blind operator. A discrimination index (DI) was calculated for the novel object, defined as the amount of time spent exploring the novel object over the total time spent exploring both objects × 100 [DI = tN/(tN + tF) × 100].

Following NORT, mice were assessed in the Y maze. The Y maze evaluate short-term working memory [29, 30] and was constructed with three black Plexiglas arms at equal angles (30 cm length, 12 cm width, and 5 cm height). Each mouse was allowed to explore freely in the Y-maze for 8 min. A triad was identified as a set of three different arm entries. The number of triads and arm entries were recorded. The alternation percentage was calculated by [the number of triads/(the number of possible alternations-2)] × 100.

Morris water maze (MWM) is to test hippocampal-dependent and long-term spatial memory, including the acquisition of spatial (hidden-platform) and non-spatial (visible platform) memory [31]. For the entirety of the assay, visual cues and ambient lightning were kept constant. The training phase with a hidden platform consisted of 4 × 60 s attempts per day, for 5 consecutive days. The trial was considered a success if the mouse found the platform within 60 s and stayed there for 5 s. If the mouse failed to reach the escape platform after 60 s, the mouse was guided to the platform and allowed to stay there for 10 s before being taken out of the tank. On the testing sixth day, the submerged escape platform was removed. For each day of training, escape latency was averaged across four daily trials.

#### Emotional behavioral tests

And then the elevated plus maze (EPM). EPM is an unconditioned reflex model based on the animal’s spontaneous fear-like reaction [32, 33]. The EPM consists of two open arms (30 × 8 cm) and two closed arms (30 × 8 × 15 cm) connected with a central area (8 × 8 cm). The mouse was placed in the central area with its head facing the open arm, then the number of entries the mouse enters the open arms and the residence time in the arms within 5 min were recorded.

The last behavioral test is forced swimming test (FST) [34, 35]. FST was widely used in screening of potential antidepressants, and it is also one of the most commonly used experiments to evaluate depression behaviors in rodent models. The mouse was introduced in a 30-cm-depth cylindrical container filled with 22 ± 1 °C clean water and forced to swim. The immobile state means that the animal gives up actively struggling, with the nose on the water, and the body shows passive floating, or no twisting. Each test lasted for 6 min, but the duration of immobility was only recorded for the latter 4 min.

### Brain collection for pathology

Animals were deeply euthanized and perfused with 0.9% saline. Brains were dissected, and the two hemispheres separated, with one post-fixed in 4% paraformaldehyde at 4 °C for 24 h, followed by dehydration. The second hemisphere was quickly micro-dissected to isolate the cerebral cortex and hippocampus (containing the injection site), and was stored at – 80 °C for subsequent use in biochemistry experiments.

### Thioflavin S staining

For fibrillar Aβ staining, brain sections were firstly washed in PBS for 3 times and 5 min each time, then washed in ddH_2_O twice and 5 min each time. Later incubate brain sections in 1% thioflavin S (Sigma-Aldrich) for 5 min, then color separation in 70% ethanol and mounted with mounting medium (Vector Laboratories) when the ethanol is almost dry. Fluorescent dyes were imaged using an Olympus microscope. The size of Aβ plaque in each field was calculated and 6 microscopic fields of each section were examined.

### Nissl staining

Sections were mounted onto microscope slides and then bathed for 15 min in a prewarmed solution of 0.1% cresyl violet. After removing the excess dye with water for 3 min, sections were destained in 95% ethanol for 5 min and dried. Sections were then washed twice in xylene for 5 min and coverslipped with neutral balsam. Neuronal loss was quantified using ImageJ software in a blinded manner.

### Thioflavin S/IHC/hematoxylin multiple staining

After completion of IHC DAB labeling, the slides were allowed to dry at RT for 12 h, rehydrated, and immersed in hematoxylin solution for 5 min. Finally, the slides were incubated with 1% Thioflavin S in 70% ethanol for 10 min, rinsed in 70% ethanol for 5 min and sealed with fluorescent mount medium [36]. The microglia numbers per Aβ plaque were measured.

### Immunohistochemistry and immunofluorescence

The mice’s brain tissues were embedded in OCT and sliced into 30 μm coronal sections on a frozen section machine (Leica, Germany). The brain slices were collected sequentially and stored at – 20 °C. Per frozen sections were antigen-retrieved in pH 6.0 citric acid antigen retrieval solution for 5 min and washed in 0.01 M PBS. After treatment with 3% hydrogen peroxide and permeabilizing using 0.3% Triton X-100, the sections were blocked in normal goat serum solution and then were incubated overnight at 4 °C with primary antibodies: anti-Tβ4 antibody (1:200, Abcam, ab167650), anti-Aβ_1-42_ antibody (1:800, NOVUS, NBP2-13075), anti-Iba1 antibody (1:1200, GeneTex, GTX100042), anti-iNOS antibody (1:400, Proteintech, 18985-1-AP), anti-CD206 antibody (1:200, Proteintech, 60143-1-AP), anti-GFAP antibody (1:3000, Novus, NB300-141), anti-S100β antibody (1:400, Proteintech, 15146-1-AP), anti-MAP2 antibody (1:400, Proteintech, 17490-1-AP), anti-TNF-α antibody (1:400, Novus, NBP1-19532), anti-NeuN antibody (1:200, Millipore, MAB377), or anti-5-HT_1A_R antibody (1:300, GeneTex, gtx100329). The specificity of this antibody has been verified by the manufacturer. The next day, the corresponding biotinylated secondary antibodies were used. The last step was DAB staining and fluorescent mounting medium DAPI (Vector Laboratories). Six random visual fields of the cortex or hippocampus were photographed in each section.

### Morphological analysis of marker-labeled cells

There were 3 biological replicates in per group for Thioflavin S and there were 4 biological replicates in per group for Nissl staining, IHC, and IF staining index. For q-PCR, ELISA, and western blot, we used 5 replicates per index, and 3 technical replicates were done in each test. For Thioflavin S staining analysis, images of fluorescent field at × 10 magnification were produced. For other staining, slices in bright field or fluorescent field at × 20 or × 40 magnification were produced. The marker-labeled area and cells were examined in PtA and hippocampus. The plaque size was obtained by manually tracing its perimeter. To quantify the positive stained area, 4–10 representative images of each region were analyzed and quantified by ImageJ software (National Institutes of Health (NIH), Bethesda, MD, USA) as following steps: (1) images are converted to 8 bit for the quantify images; (2) following converting, images are thresholded for area of marker-positive cells and background signals are removed; (3) regions of interests (ROIs) drawn for individual brain sections were then analyzed for total tissue area; (4) thresholded images for the designated brain area are quantified by calculating staining area or optical intensity relative to the total area of the analyzed region; (5) to normalize relative to the control. The immunoreactivity was represented by value of AOD (average optical density) or mean fluorescent density. The number of marker-labeled cells was manually counted using ImageJ in a blinded manner, the statistical index was the average density (number/mm^2^) of 6 images for each subregion (× 3 sections). Cells (25–35) were analyzed in each brain slice for Iba1 AOD levels and microglia activated or deactivated state. Microglia morphologies can be categorized descriptively and quantified as a continuous variable for parameters such as cell ramification, complexity, and shape [37]. The morphology analysis requires marker-labeled cells intact and unobscured by other cells or background labeling, then obtained semi-automatically with the Sholl analysis [38, 39] analyzed with ImageJ software (NIH, Bethesda, USA). The numbers of activated or deactivated microglia were manually counted. The criteria for activated microglia referred to the descriptions of Diz-Chaves et al. (2012) [40] and Roque et al. (2016) [41], in which microglia were classified into 5 morphological types. Types I–III (small soma size and few to numerous processes) were defined as resident microglia; types IV–V (large soma size or amoeboid body, and thicker and short processes) were defined as activated microglia. The mean value was obtained by averaging the counts of three coronal sections for each mouse.

### Western blotting

The proteins of mice cortex and hippocampus were then examined by western blotting. Before use, tissues were homogenized in lysis buffer I made of HEPES, MgCl2, KCl, supplemented with protease inhibitor (Targetmol, L1100 ), and phosphatase inhibitor (Targetmol, T4671) cocktails on ice, and then centrifuged at 16000×*g* at 4 °C for 30 min. Supernatants were collected and stored at − 80 °C as the cytoplasmic protein. The pellets were resuspended in lysis buffer II made of HEPES, MgCl2, KCl, EDTA and glycerinum, supplemented with phosphatase and protease inhibitor cocktails, followed by lysis on ice for 30 min. The homogenates were then added with lysis buffer III made of HEPES, EDTA, phosphatase, and protease inhibitor cocktails, then centrifuged at 16000×*g* at 4°C for 30 min at 4 °C. And the supernatants recovered as the nucleoprotein and stored at − 80 °C. A BCA protein assay (Xian Heart Biological Technology Co., Ltd, WB003) was used to determine protein concentrations. Then, 20 μg of extracts were loaded onto 10% SDS-PAGE gels. Following electrophoresis, proteins were transferred to a polyvinylidene difluoride (Millipore) membrane. Membranes were then blocked for 1 h in 5% nonfat milk in Tris-buffered saline containing 0.1% Tween 20 (TBST) at room temperature. The membrane were incubated overnight at 4 °C with the following primary antibodies: GluR1, Synapsin1, PSD95, TLR4, TLR2, TNFR2, MyD88, IKK-β, IKB-α, NF-κB p65, phospho-NF-κB p65(Ser536), NF-κB p52 (Cell Signaling Technologies), 5-HT_1A_R (GeneTex), GAPDH, β-actin, and α-tubulin(Proteintech group), followed by incubation with an HRP-conjugated secondary antibodies for 2 h at room temperature. An ECL Detection Kit (Fdbio-Femto ECL solution, FD8030) was used to detect immunoreactive proteins. The immunoblotting images were captured, intensities of bands were quantified and normalized using the corresponding signal for internal control proteins using ImageJ software.

### ELISA

The cortex and hippocampus were added with equal volume of RIPA buffer containing protease inhibitors, sonicated briefly, and lysed on ice for 30 min with gentle agitation. Debris was then removed by centrifugation. Aliquots of the lysates were removed and assayed for Aβ_1-42_ using Human Aβ_1–42_ enzyme-linked immunosorbent assay (ELISA) kits (R&D Systems, USA) according to the manufacturer’s instructions.

### qRT-PCR

Total RNAs from the mouse brain cortex and hippocampus were extracted with MiniBEST Universal RNA Extraction Kit. RT of total RNA to cDNA was carried out with the PrimeScript RT Master Mix. Real-time PCR was performed to measure mRNAs using TB Green Premix Ex Taq II in a fluorescence thermocycler iQ5 Thermal Cycler (Bio-Rad) following the manufacturer’s instructions. The three kits were obtained from Takara Biotechnology Corporation (Dalian, China). The primer sequences are shown in Table 1 (at the bottom of the manuscript). Relative gene expression levels were calculated using 2^−ΔΔCT^ mathematical model.

**Table 1:** The primer sequences used in this experiment

Gene	Accession number	Primer sequence
TLR4	NM_021297.3	M-TLR4-P1 5-CGGAAGGTTATTGTGGTAGT-3
		M-TLR4-P2 5-CTGCTAAGAAGGCGATACA-3
TLR2	NM_011905.3	M-TLR2-P1 5-CTGTTGATCTTGCTCGTA-3
		M-TLR2-P2 5-GAATCCTGCTCACTGTAG-3
TNFR2	NM_011610.3	M-TNFR2-P1 5-CAACTCTAAGTGCCATCC-3
		M-TNFR2-P2 5-CTCCAACAATCAGACCAAT-3
IL-1β	NM_008361.4	M-IL1β-P1 5-CTTCAGGCAGGCAGTATC-3
		M-IL1β-P2 5-CCAGCAGGTTATCATCATCA-3
IL-10	NM_010548.2	M-IL10-P1 5-GGTTGCCAAGCCTTATCG-3
		M-IL10-P2 5-TCCACTGCCTTGCTCTTAT-3
YM1	NM_009892.3	M-YM1-P1 5-CGTCAGATATTCATTCAGTCAGTTA-3
		M-YM1-P2 5-GTGAGTAGCAGCCTTGGA-3
Fizz1	NM_020509.4	M-FIZZ1-P1 5-GAACTTCTTGCCAATCCA-3
		M-FIZZ1-P2 5-GTCCAGTCAACGAGTAAG-3
Tβ4	NM_021278.2	M-Tbeta4-P1 5-GTCTGACAAACCCGATATGG-3
		M-Tbeta4-P2 5-GCCAGCTTGCTTCTCTTG-3
β-actin	NM_007393.3	M-actin-P1 5-ACCACACCTTCTACAATGAG-3
		M-actin-P2 5-ACGACCAGAGGCATACAG-3
TNF-α	NM_013693.3	M-TNFa-P1 5-CCTATGTCTCAGCCTCTT-3
		M-TNFa-P2 5-GAACTTCTCATCCCTTTGG-3
TGF-β	NM_011577.2	M-TGFb-P1 5-GCAACAACGCCATCTATG-3
		M-TGFb-P2 5-AAGGTAACGCCAGGAATT-3

### Statistical analysis

Analysis was carried out with IBM SPSS Statistics 20.0 and GraphPad Prism (v6.0). All data were expressed as mean ± SEM. The escape latency during the spatial learning tests was determined by a two-way repeated-measures analysis of variance (ANOVA) with LSD post hoc tests. All other data were analyzed by one-way ANOVA with Tukey-Kramer post hoc tests. Differences were deemed to be significant if *p* < 0.05.

## Results

### Persistent over-expression of Tβ4 mediated by a lentiviral vector increased its levels in the mouse brain

**Fig. 2:**
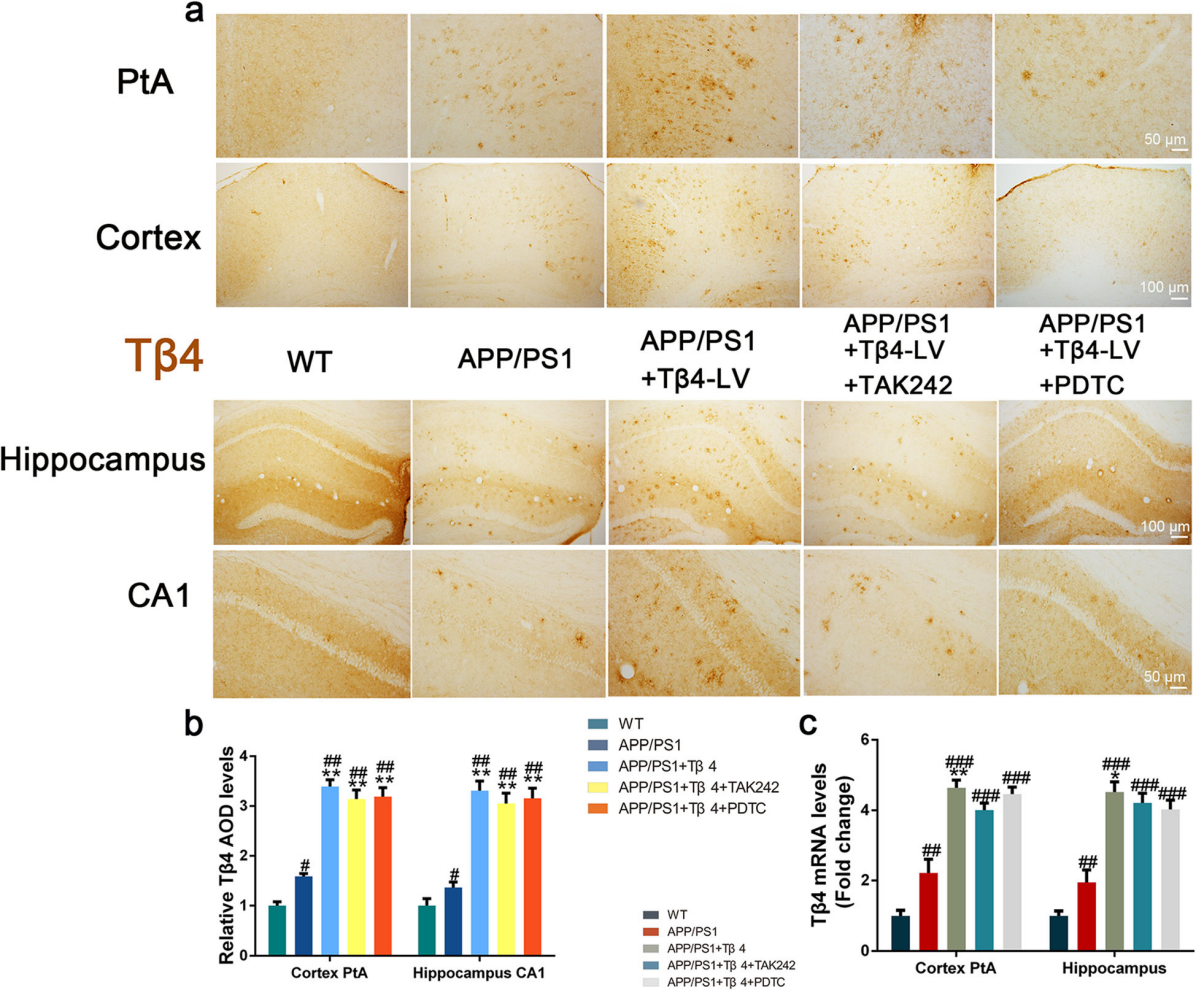
Tβ4 expression in the cortex PtA and hippocampus CA1 in each group of mice. **a** Representative images showed Tβ4 staining in the cortex PtA and CA1. **b** Quantification of Tβ4 immunopositivity in the cortex and hippocampus of mice. The data are presented as mean ± SEM (*n* = 4/group) in the different experimental groups. **c** Tβ4 mRNA levels are shown in the cortex PtA and CA1. The data are presented as mean ± SEM (*n* = 4/group) in the different experimental groups. #*p* < 0.05, ##*p* < 0.01, ###*p* < 0.001 vs WT mice; *p < 0.05, **p < 0.01 vs APP/PS1 mice

Lentiviral vector is a valuable tool for mediating gene transfer and prolonged gene expression in non-dividing cells such as neurons [42]. The biodistribution of lentivirus was examined 4 months poststereotaxic delivery in the PtA and CA1 of mice. We observed that the GFP expression was in several brain areas both proximal and distal to the injection sites, highlighting its efficiency (Fig. 1b). The histochemistry positive staining of Tβ4 was evenly distributed in the cortex and hippocampus of 12-month-old wild-type mice, with only a fuzzy, light-colored outline. The positive staining of APP/PS1 mice showed a slight increase in optical density. While Tβ4 lentiviral transduction mice showed a stronger positivity and an increase in the distribution area in the cortex and hippocampus, with overt morphology of cells, caused a 58.75% increment in Tβ4 protein level compared to APP/PS1 mice (Fig. 2a, b). The elevation of Tβ4 expression was also evidenced by the fact that Tβ4 mRNA level augmentation (Fig. 2c).

### Aβ_1-42_ accumulation was reduced following Tβ4 gene transfer in APP/PS1 mice

**Fig. 3:**
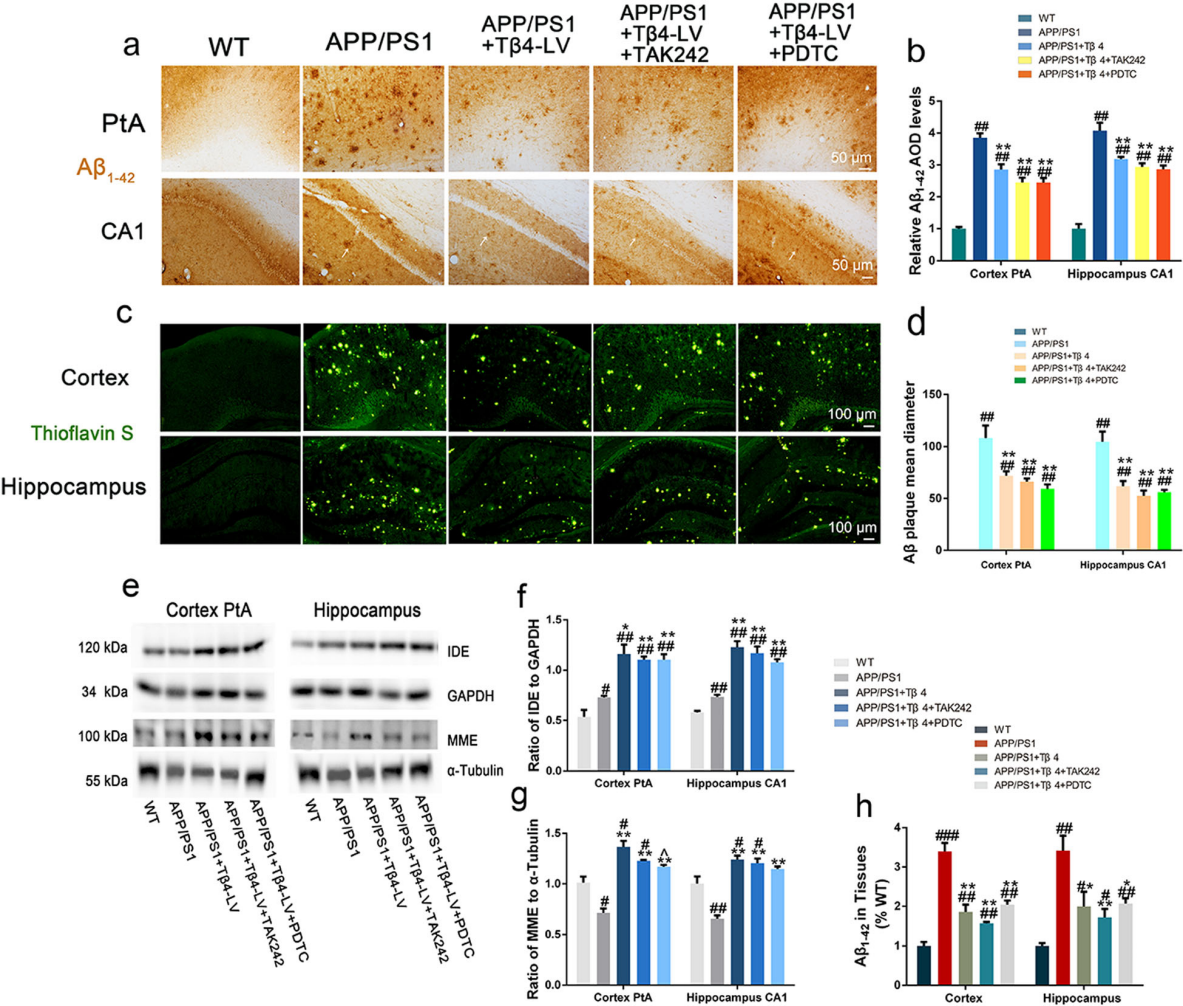
Aβ_1-42_ and fibrillar Aβ in the cortex PtA and hippocampus in groups of mice. **a** Representative images showed Aβ_1-42_ staining in the cortex PtA and hippocampus subregions. **b** Quantification of Aβ_1-42_ immunopositivity in the cortex and hippocampus of mice. The data are presented as mean ± SEM (*n* = 4/group) in the different experimental groups. **c** Representative merged images showed Aβ plaque stained by Thioflavin S in the cortex. **d** Quantification of Aβ plaque number and diameter in the cortex. The data are presented as mean ± SEM (*n* = 3/group) in the different experimental groups. **e** The protein levels of Aβ degrading enzymes IDE and MME in the cortex and hippocampus were measured by western blotting. The measurement of gray intensity of IDE **f** and MME **g** in the cortex and hippocampus. The data are presented as mean ± SEM (*n* = 5/group) in the different experimental groups. **h** Aβ_1-42_ in tissue lysis of cortex and hippocampus using ELISA detection. The data are presented as mean ± SEM (*n* = 4/group) in the different experimental groups. #*p* < 0.05, ##*p* < 0.01, ###*p* < 0.001 vs WT mice; *p < 0.05, **p < 0.01 vs APP/PS1 mice; ^*p* < 0.05 vs APP/PS1 + Tβ4 mice

We probed Aβ_1-42_ expression in the brain by immunostaining. The Aβ_1-42_ positive staining of 12-month-old wild-type mouse only showed the shallow-coloring glia-like outline, no plaque appeared. Strikingly, staining of 12-month-old APP/PS1 mice brain showed substantial formation of dark patches with large area and diameter. Compared with the APP/PS1 group, the Tβ4 intervention group had significantly reduced staining area and intensity, with looser texture. After adding TAK242 or PDTC intervention, neither Aβ_1-42_ immunoreactive area nor intensity was significantly altered by densitometric analysis, relative to Tβ4 intervention alone (Fig. 3a, b).

Similarly, the separate detection of fibrous Aβ using Thioflavin S method and Aβ_1-42_ monomers using ELISA kits exhibited the same tendency in each group (Fig. 3c, d, h). Thus, we further assessed the Aβ processing enzymes, IDE, and MME. Here too, the results indicate an increase in expression of IDE protein levels in Tβ4, TAK242 and PDTC intervention group compared with APP/PS1 group (Fig. 3e–g). Taken together, in relationship to these measurements, the Aβ reduction in the Tβ4 intervented APP/PS1 brain might relate to increased Aβ degrading enzyme levels or restored impaired clearance of Aβ.

### Lentiviral Tβ4 intervention restrained microglia infiltration

**Fig. 4:**
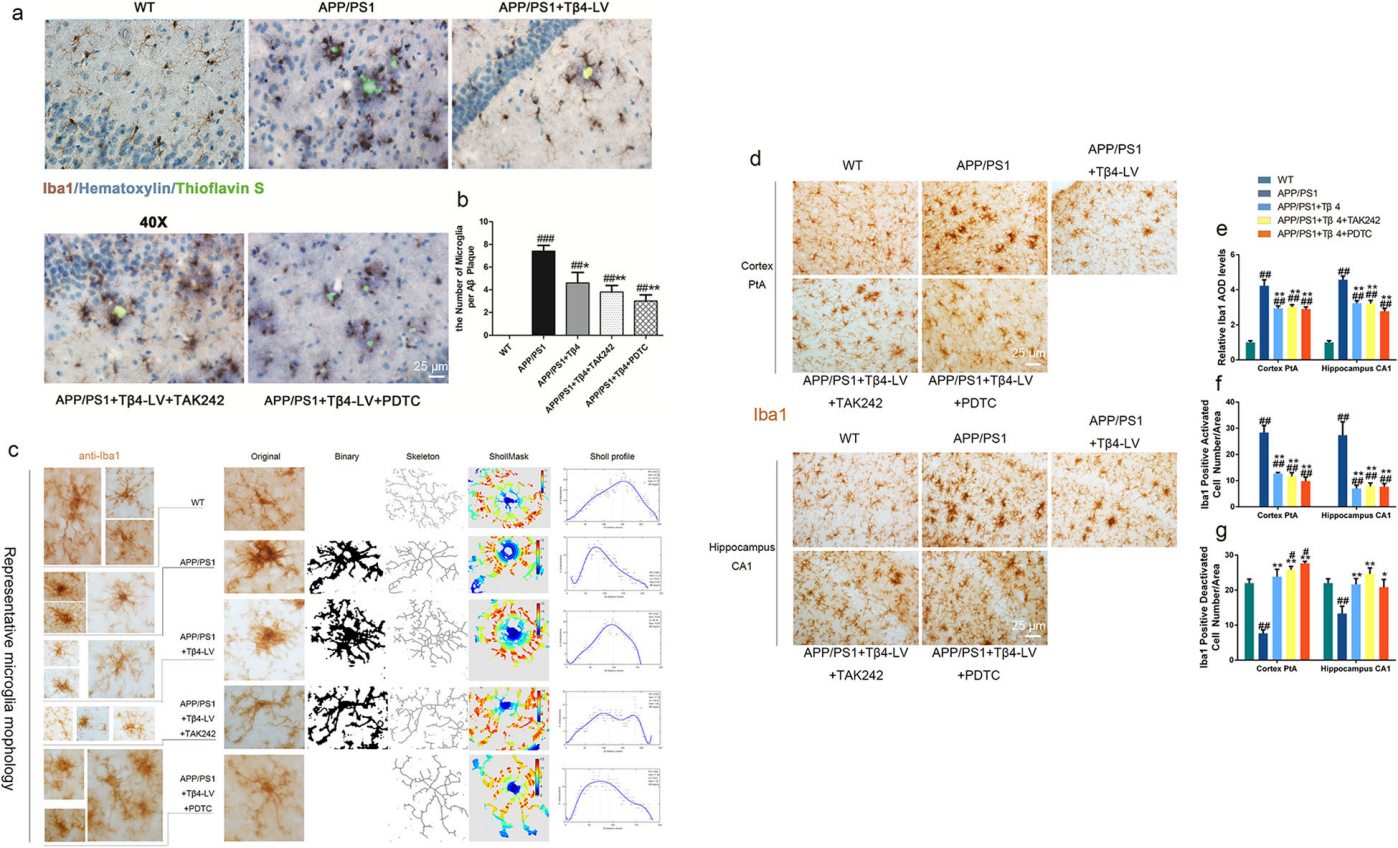
The effects of Tβ4 intervention and inflammatory pathway inhibition on microgliosis in the PtA and CA1 in groups of mice. **a** Representative images showed microglia infiltration implicated by Thioflavin S/IHC/Hematoxylin multiple staining in groups of mice. **b** Quantification of microglia surrounding per Aβ plaque. The data are presented as mean ± SEM (*n* = 4/group) in the different experimental groups. **c** Representative magnified profile of Iba1-labeled microglia morphology and process intersections by Sholl profile analysis in brain injection filed. **d** Representative images of Iba1-labeled microglia in PtA and CA1; quantification of **e** Iba1 immunopositivity, **f** number of Iba1-labeled activated and **g** deactivated microglia. The data are presented as mean ± SEM (*n* = 4/group) in the different experimental groups. #*p* < 0.05, ##*p* < 0.01 vs WT mice; *p < 0.05, **p < 0.01 vs APP/PS1 mice

Microgliosis is reported as an essential feature of neuroinflammatory response in AD [43]. We adopted a method of Iba1, hematoxylin, and Thioflavin S multiple staining to test whether the chronic neuroinflammation known to occur with AD was altered in the presence of Tβ4. We found that Aβ plaques were surrounded by activated microglia in the cortex and hippocampus of APP/PS1 mice. Compared with APP/PS1 group, the activated microglia juxtaposed to Aβ plaques in the same area in Tβ4 intervention group, TAK242, or PDTC intervention group were significantly reduced, and there was no significant difference between the three groups (Fig. 4a, b).

For Iba1 staining, we found that the microglia of wild-type mice were sparsely distributed in the cortex and hippocampus, appeared only lightly stained, with delicate and clear profile. The soma extends long process and highly complex, rarely overlapping branches. Microglia in APP/PS1 mice showed enlarged and deepened staining cell bodies, and shortened protrusions, generating a radial or echinosphered shape. While microglia with complex branches were scattered among the activated microglia clusters. Comparatively to the APP/PS1 group, microglia in the Tβ4 intervention group, the additional TAK242 and the PDTC intervention group all displayed a stark reduction in activated forms, while an increase in cell processes and the proportion of deactivated forms in the perilesional zone of brain injection. But the results among the three groups were not significantly different (Fig. 4d–g).

This also applied to Sholl analysis quantification when we summarized the represented microglia profile. Microglia in APP/PS1 mice were less ramified, amoeboid in shape, suggesting that they had become reactive, a sign consistent with the adoption of a phagocytic phenotype [44], suggesting that cells have been activated by genetically driven plaque formation in this case. However, microglia in the Tβ4 intervention group, the additional TAK242 and the PDTC intervention group showed increased intersections and more ramified, intermediates changes, a morphology that is consistent with transforming to baseline quiescent state (Fig. 4c).

### Lentiviral Tβ4 intervention reversed the phenotypic polarization of microglial

**Fig. 5:**
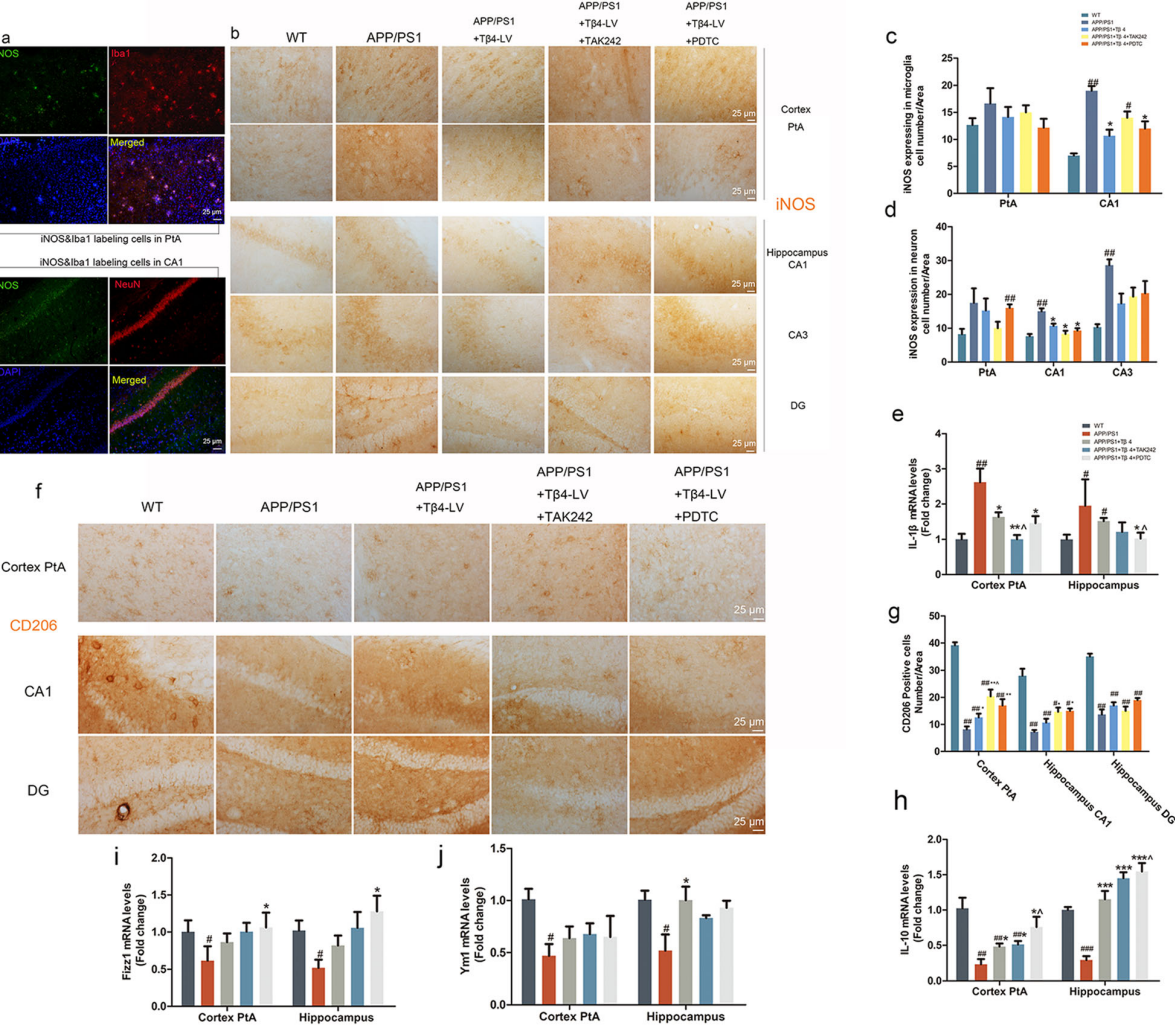
M1-phenotype and M2-phenotype of Microglia in all groups. **a** Representative photomicrographs of iNOS and Iba1, iNOS and NeuN double labeling cells in APP/PS1 mice. **b** Representative photomicrographs of iNOS-labeled cells morphology. **c** The number of microglia expressing iNOS and **d** neuron expressing iNOS in PtA and hippocampus of all groups. The data are presented as mean ± SEM (*n* = 4/group) in the different experimental groups. **e** The mRNA levels of IL-1β in cortex and hippocampus. The data are presented as mean ± SEM (*n* = 4/group) in the different experimental groups. **f** Representative photomicrographs of CD206 positive cells and **g** the number of positive cells in PtA and hippocampus of all groups. **h** IL-10, **i** Fizz1, and **j** YM1 mRNA levels in PtA and hippocampus. The data are presented as mean ± SEM (*n* = 4/group) in the different experimental groups. #*p* < 0.05, ##*p* < 0.01 vs WT mice; *p < 0.05, **p < 0.01, ***p < 0.001 vs APP/PS1 mice

To provide phenotypic polarization evidence, we used antibodies directed against iNOS, a marker of M1-phenotype, and CD206, a marker of M2-phenotype. We observed iNOS^+^Iba1^+^ cells and iNOS^+^NeuN^+^ cells as is shown in Fig. 5a. The number of iNOS^+^ cells in brain of APP/PS1 mice was significantly higher than that of the wild-type group. The number of iNOS^+^ cells in the CA1 in the Tβ4 intervention group, TAK242 or PDTC intervention group was significantly lower than APP/PS1 group (Fig. 5b–d). And IL-1β mRNA level shares the same trend (Fig. 5e). As expected, the CD206^+^ cells showed an almost opposing direction of differentiation to that of iNOS (Fig. 5f, g). This meant that Tβ4 promoted M1-phenotype transformed to M2-phenotype. Furthermore, the mRNA levels of IL-10 and Ym1 detected in the hippocampus of Tβ4 intervention mice were significantly increased than that of APP/PS1 mice, suggesting a restoration of microglia inner homeostasis, and Fizz1 mRNA level also showed a trend of elevation (Fig. 5h–j). These results demonstrated that to a certain extent, persistent microgliosis was inhibited by the predisposition of Tβ4.

### Lentiviral Tβ4 intervention suppressed astrocyte proliferation and converted its phenotype differentiation

**Fig. 6:**
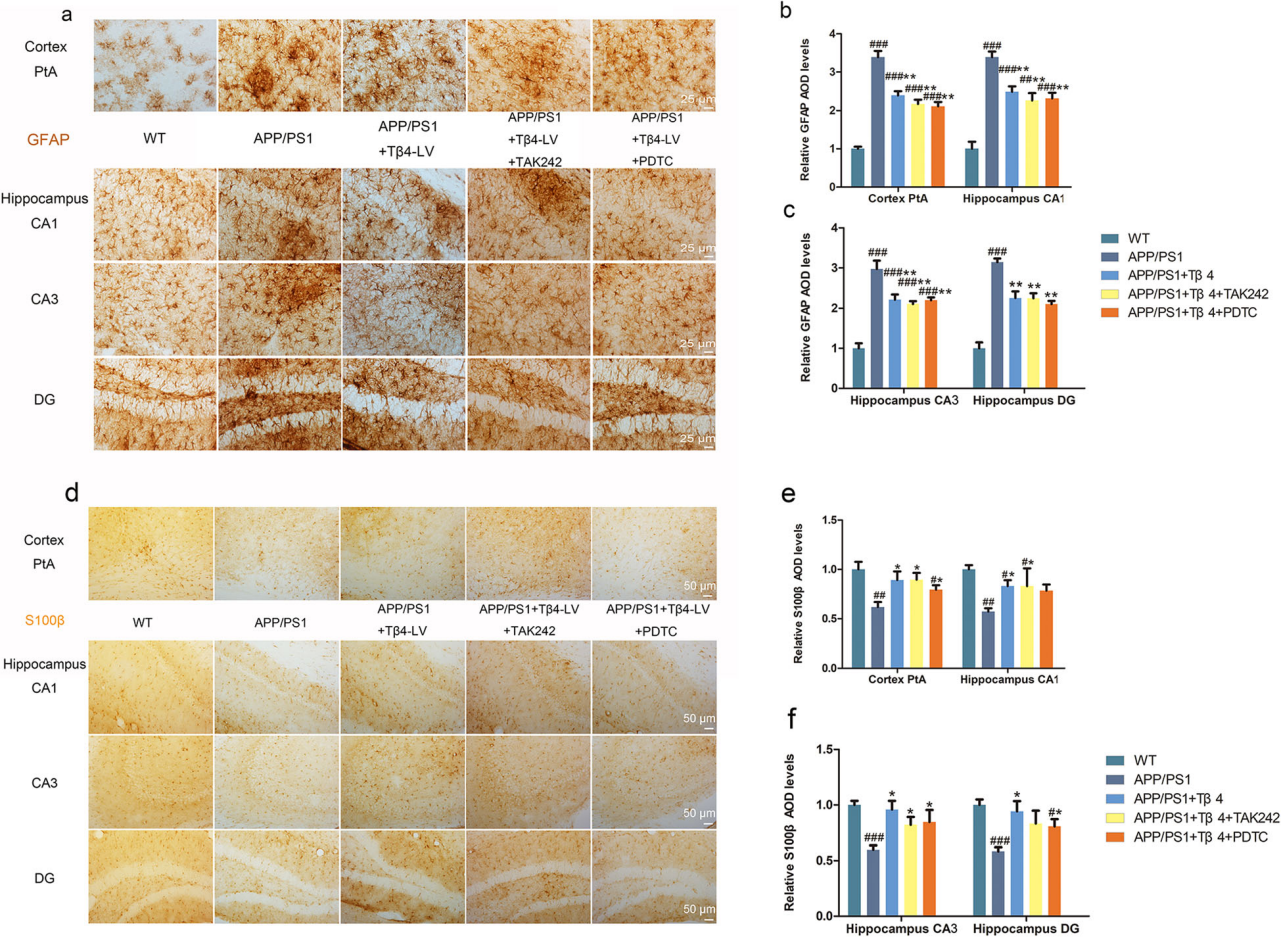
The effects of Tβ4 intervention and inflammatory pathway inhibition on astrogliosis and A2-phenotype of astrocyte in all groups of mice. **a** Representative images of GFAP-labeled astrocyte in PtA and hippocampus. **b** GFAP immunopositivity of PtA, CA1, and **c** CA3, DG region. The data are presented as mean ± SEM (*n* = 4/group) in the different experimental groups. **d** Representative photomicrographs of S100β positive cells. **e** S100β immunopositivity of PtA, CA1, and **f** CA3, DG region. The data are presented as mean ± SEM (*n* = 4/group) in the different experimental groups. #*p* < 0.05, ##*p* < 0.01, ###*p* < 0.001 vs WT mice; *p < 0.05, **p < 0.01, ***p < 0.001 vs APP/PS1 mice

According to Liddelow et al., A1-phenotype of astrocytes (unhealthy astrocytes) highly upregulate many classical complement cascade genes to be destructive to synapses. In contrast, A2-phenotype of astrocytes (healthy astrocytes) release many neurotrophic factors and regulate brain homeostasis [45]. We found that there was an enhancement of GFAP expression in APP/PS1 mice, with the GFAP-positive astrocytes forming large obvious glial scars. In Tβ4, TAK242, or PDTC intervention group, the reactive hyperplastic colloidal scar was restricted, with the hippocampal DG area the most significant (Fig. 6a–c). S100β is a marker of A2-phenotype. Compared with the APP/PS1 group, the Tβ4 intervention group showed a significant S100β staining intensity increase around injection site, among which, the effect of TAK242 additional intervention was more obvious than that of PDTC in CA1. Combined with results from microglia, lentiviral Tβ4 intervention alleviates neuroinflammatory response in APP/PS1 mice (Fig. 6d–f).

### Long-term Tβ4 upregulation elevated neuronal survival and function

**Fig. 7:**
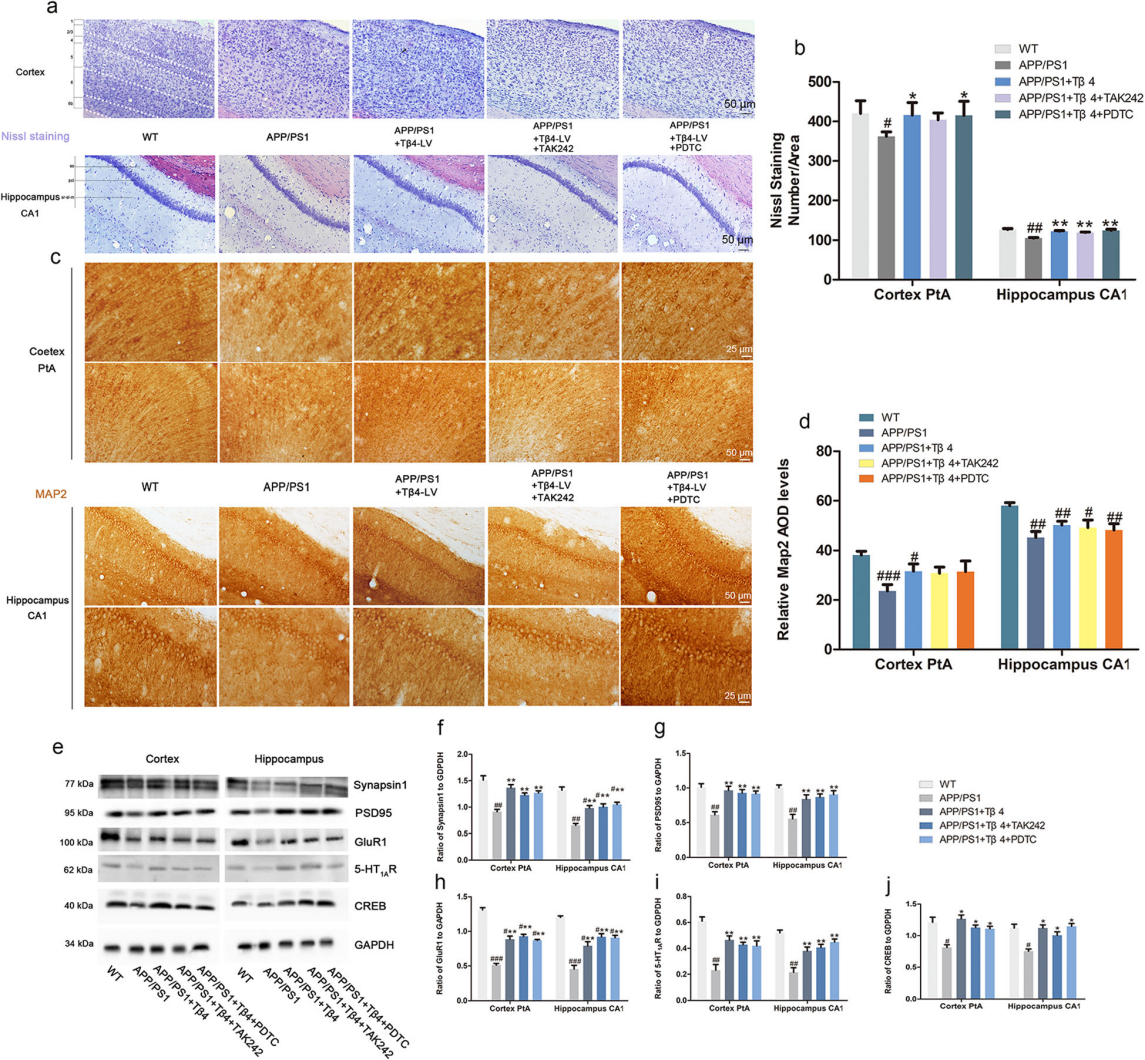
The effects of Tβ4 intervention and inflammatory pathway inhibition on neuronal survival and neurotransmitter release-related proteins. **a** Representative photomicrographs of Nissl staining. **b** Histograms are the quantitative analysis of the number of survival neurons in the PtA and hippocampus. The data are presented as mean ± SEM (*n* = 3/group) in the different experimental groups. White dashed line implicates separated cortex layers, and white dotted circle refers to neuronal loss, with black arrow pointing to the location. **c** Representative photomicrographs of Map2 DAB staining and **d** Map2 immunoreactivity in the PtA and CA1 region. The data are presented as mean ± SEM (*n* = 4/group) in the different experimental groups. **e** The protein levels of Synapsin1, PSD95, GLUR1, 5-HT_1A_R, and CREB in the cortex and hippocampus were measured by western blotting. **f**–**j** The measurement of gray intensity of these proteins in the cortex and hippocampus. The data are presented as mean ± SEM (*n* = 5/group) in the different experimental groups. #*p* < 0.05, ##*p* < 0.01 vs WT mice; *p < 0.05, **p < 0.01 vs APP/PS1 mice

Neurons are the main bearers of normal nervous system functions. As is shown in Nissl staining, the neurons of wild-type mice were morphologically normal with distinct hierarchical structure and regular arrangement of the 6 layers of cortex and 3 layers of hippocampus CA1, and rich in Nissl body particles. In APP/PS1 mice, the neurons showed a decline and derangement, with an unclear nuclei boundary. Compared with the APP/PS1 group, the Tβ4 intervention group, the addition of TAK242, and the PDTC intervention group showed restoration of the above phenomenon. The results among the three groups were not significantly different (Fig. 7a, b). Map2 staining manifested matured neurons with long and dense nerve fibers in WT mice. Interestingly, we found that Tβ4 intervention seemed to precipitate the recovery of axonal length of but not the positive intensity microscopically in APP/PS1 mice, the recovery was restricted to the injection proximal site, with the distal sites remaining unaltered (Fig. 7c, d).

**Fig. 8:**
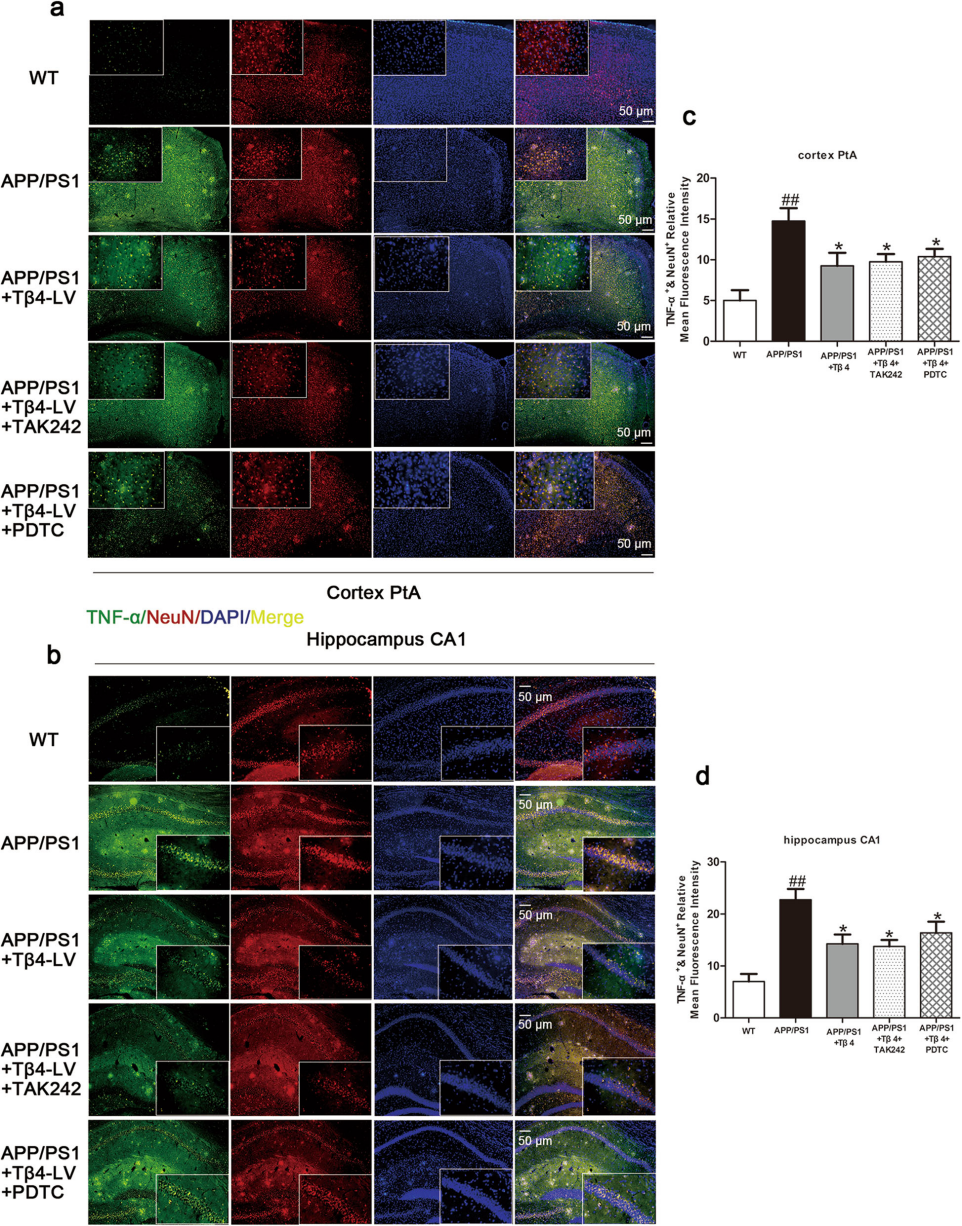
The effects of Tβ4 intervention and inflammatory pathway inhibition on TNF-α expressing in neurons. Representative photomicrographs of NeuN^+^ and TNF-α^+^ cells in the **a** PtA and **b** hippocampus. The number of NeuN^+^ and TNF-α^+^ cells in **c** cortex and **d** hippocampus. The data are presented as mean ± SEM (*n* = 4/group) in the different experimental groups. #*p* < 0.05, ##*p* < 0.01 vs WT mice; *p < 0.05 vs APP/PS1 mice

Synapsin1 was considered of importance for the regulation of neurotransmitter release [46]. AMPAR mediates most of the CNS rapid excitatory transmission and is related to the formation, stability, and plasticity of synapses [47, 48]. GluR1 is necessary for the formation of hippocampal long-term potentiation (LTP) and short-term memory [49]. PSD95 is related to experience-dependent plasticity and plays an indispensable role in learning [50]. A western blotting method was used to detect these proteins, and the results suggested that the Tβ4 treatment increased the expression of the above indicators. It implied the improved synaptic plasticity and neurotransmitter release ability of Tβ4 intervention (Fig. 7e–j). We then observed the effect of inflammatory factors on neurons in each group and found that Tβ4 reduced TNF-α^+^NeuN^+^ cells in APP/PS1 mice (Fig. 8a–d).

### Prolonged over-expression of Tβ4 improved cognitive memory and reduced the depression-like behavior in APP/PS1 mice

**Fig. 9:**
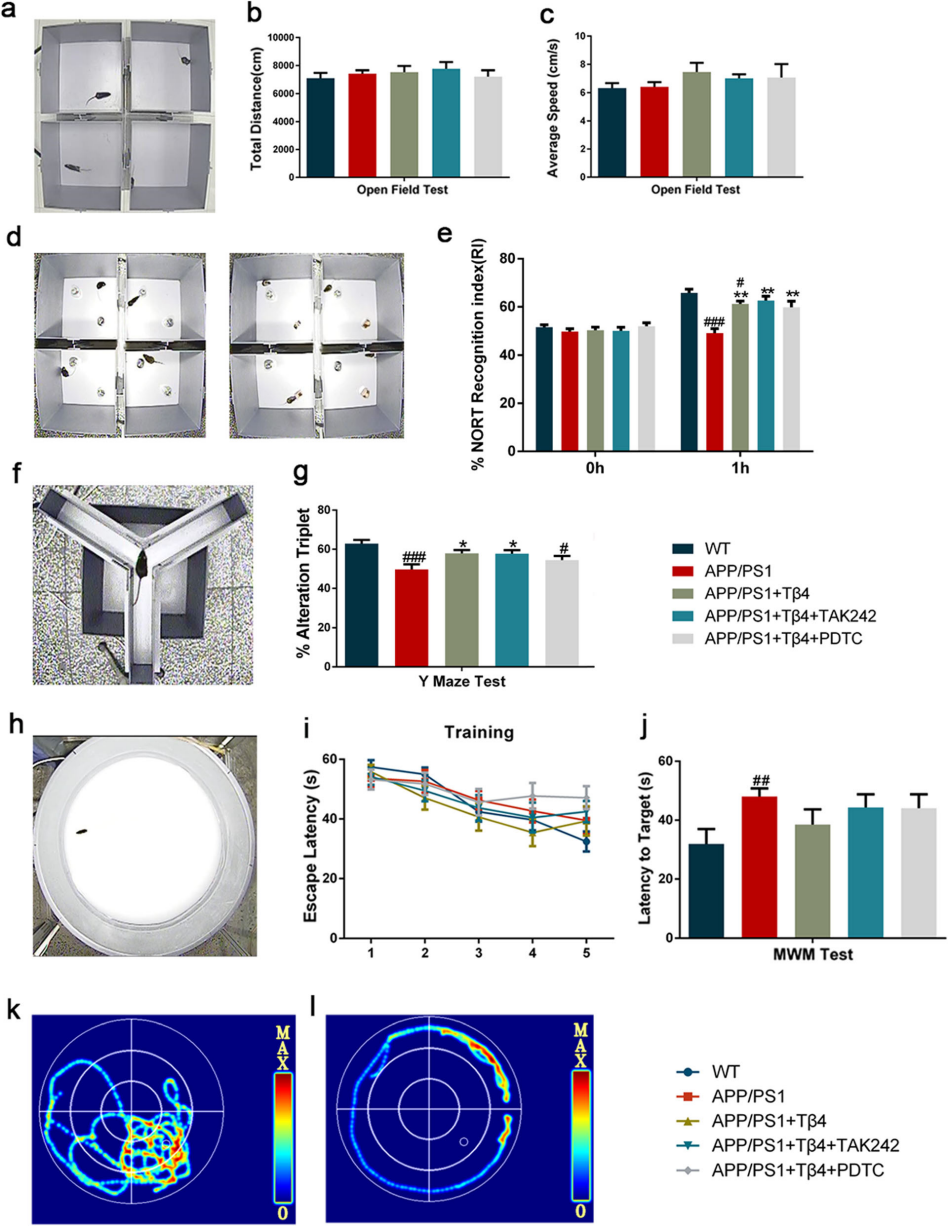
The effects of Tβ4 intervention and inflammatory pathway inhibition on mice learning and memory performance. Diagrams of **a** open field test, **d** novel object recognition test, **f** Y maze test and **h** Morris water maze test. **b**, **c** The total distance and average speed in each group. **e** The percentage of NORT recognition index were recorded to test the recognition memory. **f** The alteration triplet was recorded to test space working memory. **i** Escape latency during the training days. **j** Escape latency in the test day were recorded to assess spatial memory. Representative maps for linear (**k**) and marginal (**l**) exploring strategy. The data are presented as mean ± SEM (*n* = 11–12/group). #*p* < 0.05, ##*p* < 0.01, ###*p* < 0.001 vs WT mice; **p < 0.01 vs APP/PS1 mice

As expected, there was no difference in motor activities among all groups in the open field test (Fig. 9a–c). We found that APP/PS1 mice spent least time with the novel object, displayed an impairment of object memory retention. In opposition, Tβ4 intervention mice spent considerably more time with the novel object than the familiar one. In addition, the mice who underwent a combined treatment of TAK242 or PDTC also successfully distinguished between two different objects (Fig. 9d, e).

We extended the analysis to assess spatial working memory using Y maze test. The spontaneous alternation rate of Tβ4 intervention mice was significantly different from the AD model group (Fig. 9f, g). Then, we conducted MWM test to assess orientation learning and memory. The escape latency was longer in each group of mice in contrast to WT mice. A similar pattern was observed regardless of Tβ4 intervention in test day, learning deficits remained severe in all groups of mice. The differential effect of Tβ4 intervention on the working memory versus spatial memory should be emphasized on the hippocampal alterations (Fig. 9h–l).

**Fig. 10:**
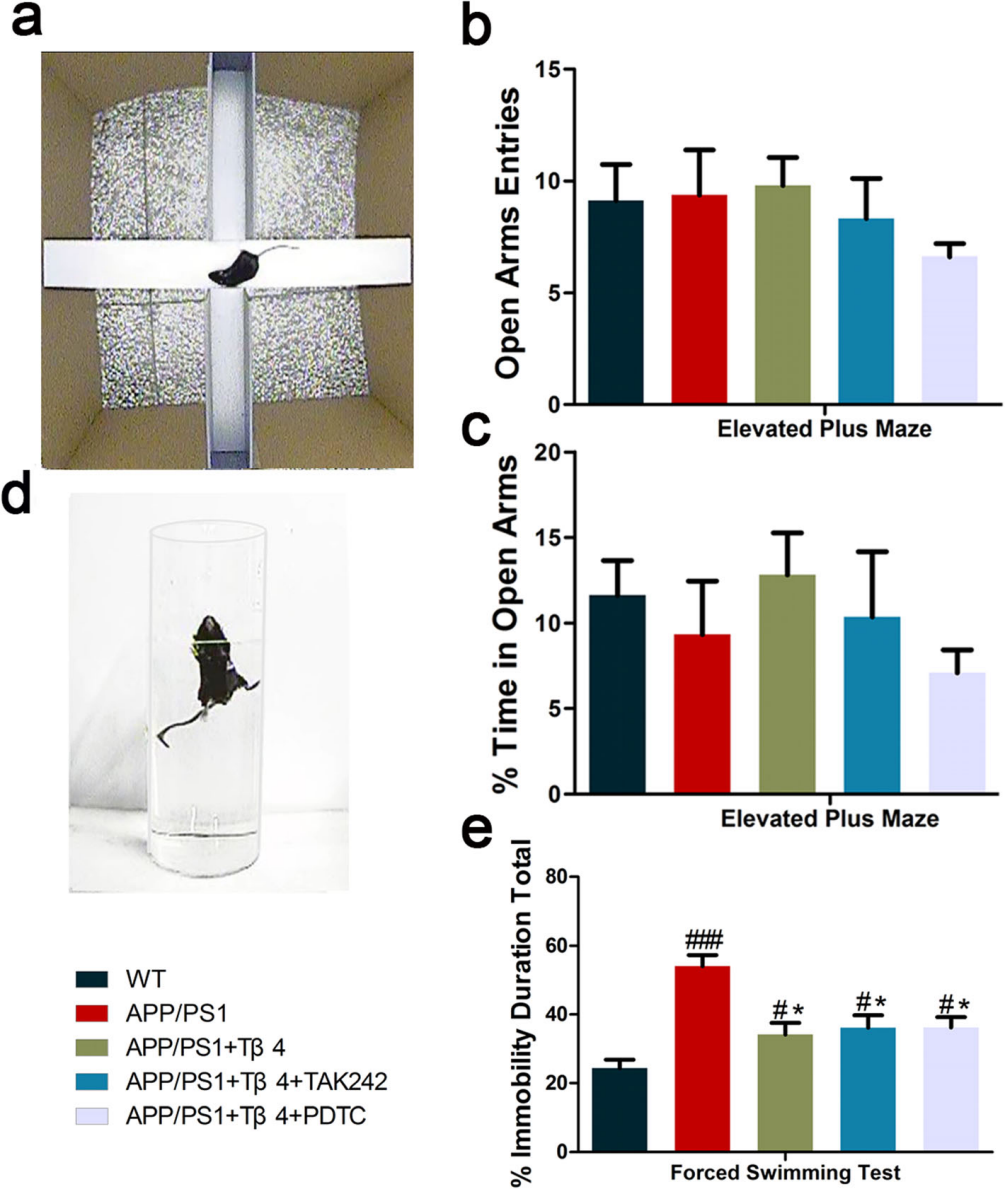
The effects of Tβ4 intervention and inflammatory pathway inhibition on mice emotional performance. Diagrams of **a** elevated plus maze and **d** forced swimming test. **b** Entries and **c** time in open arms were recorded to assess anti-anxiety-like behavior. **e** Immobility duration were recorded to test depression-like behavior. The data are presented as mean ± SEM (*n* = 11–12/group). #*p* < 0.05, ###*p* < 0.001 vs WT mice; *p < 0.05 vs APP/PS1 mice

To test whether Tβ4 intervention had a positive impact on emotion, we carried out EPM and FST after MWM. EPM test turned out that there was no obvious anxiety behavior observed in APP/PS1 mice and no significant difference in the number of open arm entries among groups (Fig. 10a–c). Dramatically, contray to EPM, the influence of Tβ4 intervention was more pronounced in shortening the immobility time of AD model mice (Fig. 10d, e). Finally, as a supplement, we investigated the 5-HT_1A_ receptor. Intriguingly, an increase in its protein level has been observed after Tβ4 intervention in comparison to APP/PS1 mice (Fig. 7e, i, j).

In short, Tβ4 showed a tendency to improve memory and emotion, especially working cognitive memory and depression. While combined blocking of TLR4 or NF-κB yielded minimal or no symptomatology rehabilitation.

### Tβ4 can negatively regulate TLR4/MyD88/NF-κB pathway

**Fig. 11:**
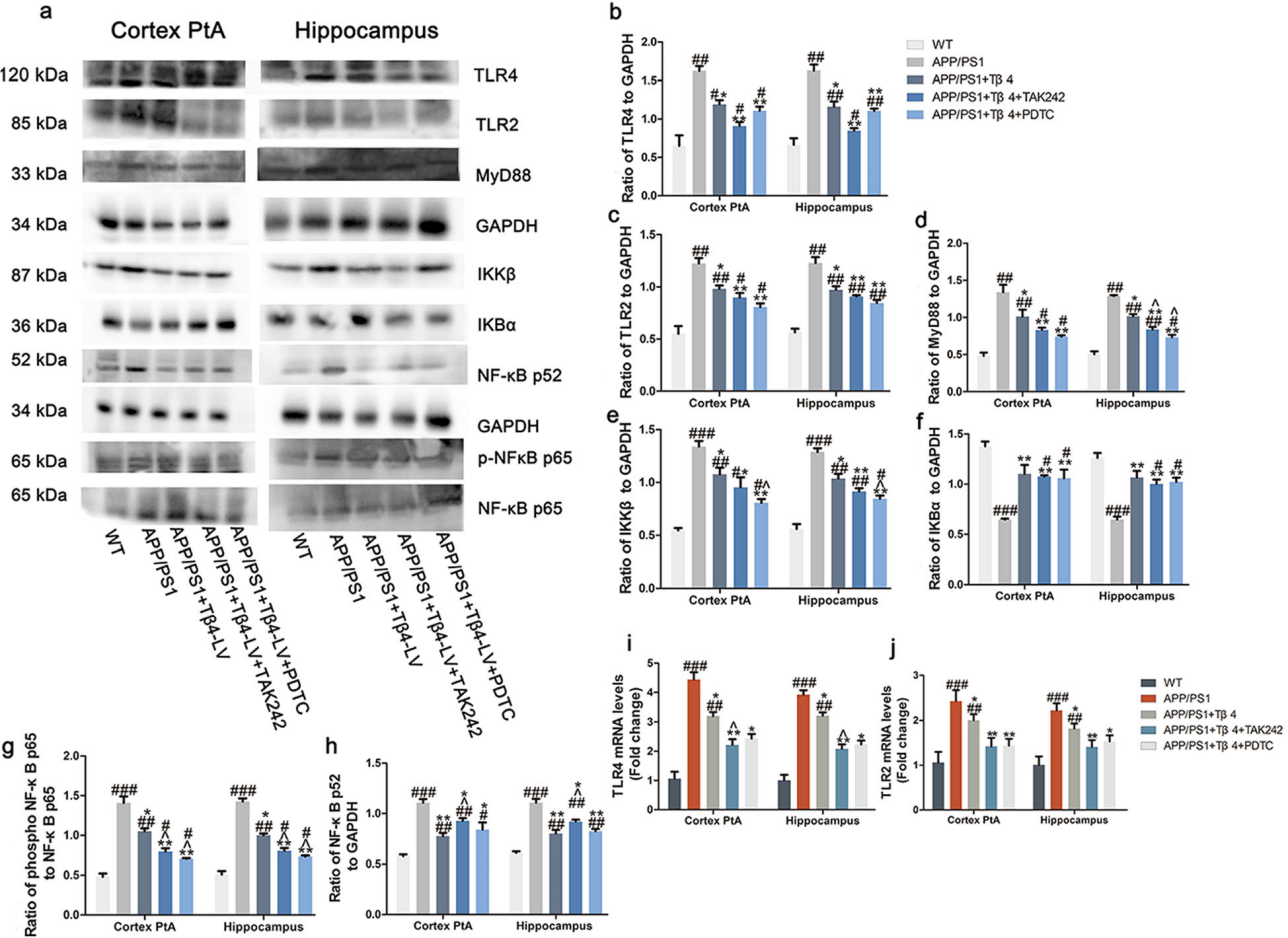
The effect of Tβ4 on the NF-κB signaling axis. **a** The protein levels of TLR4, TLR2, MyD88, IKK-β, IKB-α, NF-κBp65, phospho-NF-κBp65, and NF-κBp52 in the cortex and hippocampus were measured by western blotting. **b**–**h** Quantification of the expression of genes associated with inflammatory pathways. The mRNA levels of TLR4 (**i**) and TNFR2 (**j**). The data are presented as mean ± SEM (*n* = 5/group) in the different experimental groups. #*p* < 0.05, ##*p* < 0.01, ###*p* < 0.001 vs WT mice; *p < 0.05, **p < 0.01, ***p < 0.001 vs APP/PS1 mice; ^*p* < 0.05 vs APP/PS1 + Tβ4 mice

To discover the relevance of the observations and the potential molecular mechanisms, we emphatically examined the expression of inflammatory pathway proteins after treatments. As expected, the APP/PS1 mice brain rendered the strongest inflammatory activation, with TLR4 and TLR2 mRNA levels, TLR4, TLR2, MyD88, IKK-β, and Phospho-NF-κB p65 (Ser536)/total NF-κB p65 protein levels all increased. In addition to NF-κB p65 (NF-κB3), we probed NF-κB p52 (NF-κB2), which normally existed as inhibitory molecules in the form of precursor p100 and could not activate gene transcription, also revealing an increase. Critically, Tβ4 could suppress both the TLR4/MyD88/NF-κB p65 and NF-κB p52 inflammatory pathway molecules. These findings were supported by immunoblots and q-PCR of the cortical and hippocampal extracts. Whereas the combined TLR4 antagonist or NF-κB p65 inhibitor provided a further striking decline of TLR4 or Phospho-NF-κB p65 (Ser536)/total NF-κB p65 protein levels respectively as compared to Tβ4 intervention alone, interpreting inconsistent changes with the former observations. This might be attributed to the selectivity or specificity of the two drugs to their targets. From this, we speculated that the Tβ4 intervention alone could obtain improvement through a certain inhibition of inflammatory pathway and other hidden signaling pathways in view of the stronger effect on downregulation of TLR4/NF-κB p65 in combined TLR4 antagonist or NF-κB p65 inhibitor treatment (Fig. 11a–j).

## Discussion

The first aim of this study was to investigate whether Tβ4 intervention could create an affirmative influence on phenotypic polarization of glial cells and cognitive impairment, as well as the mechanisms underlying its ability to suppress pro-inflammatory signaling. We incidentally observed its impact on emotion. We firstly found an upregulating expression of Tβ4, TLR4, and NF-κB in APP/PS1 mice, further study showed upregulation of Tβ4 mediated the inhibitory effect of NF-κB signaling pathway activities. The major findings of the current work were as follows: over-expression of Tβ4 reduced Aβ deposition in APP/PS1 mice, inhibited glial infiltration, altered phenotypic polarization of glial cells and phenotype differentiation, improved neuronal functions, and the cognitive memory of AD model mice, accompanied by the reduced depression-like behavior of transgenic mice. And we proposed that Tβ4 may achieve these improvements mainly via negative regulating both the classic TLR4/NF-κBp65 and non-canonical NF-κBp52 inflammatory signaling pathways.

We intervened at middle age, as this was a mature stage for mice symptoms and typical pathology to fully develop in APP/PS1 mice [51]. The cortex and hippocampus are the main regions of Aβ deposition [52, 53]. To avoid inadequacy in over-expression, we opted for a 4-month lentiviral transferring [54]. The study has demonstrated good efficacy that long-term Tβ4 upregulation yielded persistent expression of the transgene in vivo. In our APP/PS1 mice brains, Tβ4 was found elevated in neuronal cells. We reasonably assumed that the rise of Tβ4 expression in APP/PS1 mice brain was a compensatory mechanism in response to reactive gliosis or may emerge as part of negative feedback leading to the deactivation of microglia and inhibition of its adverse pro-inflammatory phenotype. In Huntington’s brains, Tβ4 in the reactive microglia distributed to regions of neurodegeneration, revealing that the increase in Tβ4 during the early stages of pathology could be linked to anti-inflammatory and repair functions of microglia, since reactive gliosis is an early protective event induced by the onset of neuropathological changes [55].

Rush, T reported that Aβ oligomers rapidly induce aberrant stabilization of F-actin within dendritic spines, which impairs synaptic strength and plasticity [56]. The Aβ mediated disrupted actin dynamics or cytoskeleton remodeling even existing in early stage of AD [57, 58], leading to cognitive deficits in Alzheimer's disease. In our Tβ4 treatment, the G-actin disassembly in turn decreased Aβ deposition and increased IDE, but not MME, which is inconsistent with a study that APP/PS1 mice receiving the NEP-AAV but not IDE-AAV, showing a significant decrease in total Aβ either in the hippocampus or cortex [59].

The definition of microglia activation was primarily based on its phenotype changes. That is, the phenotype changes of microglia reflect the activation state of microglia [60]. Microglia cells are finely tuned to the physiology and pathology within their micro-domains and display a diverse range of phenotype in both subtle and gross injury [61]. Studies have shown that there are abnormalities of microglia during the whole process of AD [62]. Notably, we were able to demonstrate for the first time the systematic morphological evidence to investigate the activation status and phenotypic polarization direction of glial cells in vivo. M1-phenotype markers include iNOS (inducible nitric oxide synthase), which is only induced to express when the cells are stimulated. For the study of iNOS, we also found an expression pattern in neurons, in addition to the expected microglia. This may be due to the structural similarity between the higher quantity of iNOS and nNOS (neuronal nitric oxide synthase), because oxidative stress reaction existed in the neurons during brain aging [63]. Besides, we observed an inconstancy of iNOS expression between the cortex and hippocampus. We can conjecture that hippocampus burdens considerable Aβ loads and is susceptible to oxidative stress reaction [64]. This may in part explain why the model mice showed severe and persistent spatial memory. In this study, the result that Tβ4 reduced the microgliosis and astrogliosis, and reversed the glia activated state is consistent with the effects of Tβ4 on spinal cord injury rat, which showed reduced activated microglia/astrocyte scar, decrease in pro-inflammatory cytokine gene expression and a significant increase in the mRNA levels of IL-10 [65]. Fizz1 detected in the cortex and hippocampus, Ym1 detected in the hippocampus of Tβ4 intervention mice also showed a trend of elevation (vs APP/PS1 group, *p* = 0.054, *p* = 0.052, and *p* = 0.05 separately). Due to the prominent role of the neurons in CNS, we assessed whether Tβ4 intervention could bring synaptic changes in AD. We found an improvement in the fiber sprout (Map2), synaptic plasticity (synapsin1), ability of neurotransmitter release (PSD95), and formation of normal excitatory neural network (GLUR1), the same as the other researches that Tβ4 can promote neuronal survival and neurite outgrowth in cultured spinal cord neurons [15, 66].

There are age-related changes in hippocampal actin remodeling proteins and spatial memory behavior of APP/PS1 mice [67]. In the honeybee, inhibition of actin polymerization within the brain is involved in memory formation and enhances associative olfactory memory [68]. And intra-hippocampal infusion of Tβ4 peptide fragment also increased spatial memory of C57 mice [69]. The distribution of Aβ and the neuronal expression area of Tβ4 both include neocortex, hippocampus, and amygdala. These brain areas play a key role in mediating behavioral processes, including cognitive performance, motivational processes, and fear processing. More specifically, the hippocampus, a major brain area mediating spatial navigation, memory formation, and consolidation [70], is particularly vulnerable to aging, neuroinflammation, and Alzheimer’s disease [71]. Nevertheless, in our present study Tβ4 failed to show an alteration in MWM performance in APP/PS1 mice. We believe that the behavioral differences for Tβ4 intervened mice between MWM and NOR may be due partly to the degenerative process in a middle-later stage with pathology too robust or wide-spread to be reversed. MWM tests spatial learning and long-term memory, which require complex interactions between multiple brain regions including the neocortex and the hippocampus [72], the NOR paradigm for its part evaluates episodic memory and relies more specifically on networks connecting the prefrontal cortex to hippocampus [73]. Thus, on the one hand, our observation of the most severe Aβ and neuroinflammation in the neocortex and hippocampus, might explain why the Tβ4 intervened mice still showed defects in MWM. For emotional aspects, our Tβ4 intervention acted chiefly in relieving depression. The results also showed the over-expression of Tβ4 resulted in an elevated level of 5-HT_1A_R and CREB, but how Tβ4 increased 5-HT_1A_R and relieved depression is not completely understood. The alleviated depression appears to be related to 5-HT_1A_R/PDE2/cAMP–PKA-CREB-BDNF/neuronal calcium channel phosphorylation signaling [74, 75]. G-actin was reported to form a sensor/effector apparatus for activating cAMP synthesis [76], whether there is a crosstalk between Tβ4 and postsynaptic 5-HT_1A_R signaling is worthy of further study.

Toll-like receptor family activation could promote M1 microglial activation and the activity of FPRL1 and MMP-9, both of which are involved in Aβ clearance in AD [77, 78]. So, inhibition of TLRs produces both beneficial and detrimental effects. During the preliminary experiment, we found that the expression of TLR4 in APP/PS1 mice is higher than that of TLR2, which means that it is more involved in AD, so we chose TLR4 receptor for intervention. Therefore, regulating TLR4 activation to promote Aβ clearance without inducing neuroinflammation should be a promising treatment option for AD. Tβ4 has been implicated as a potential molecular connection. Raising possibility of Tβ4’s relation to inhibition of inflammation can be linked to the high concentrations of Tβ4 found in peripheral macrophages, which shares morphological and other similarities with microglia [79] and to our previous research confirming that Tβ4 is expressed both in neurons and microglia in AβOs treated 2-month-old C57/BL mice brain [80]. In human corneal epithelial cell line HCET, Tβ4 was reported to be colocalized with transcription factor RelA/p65 in the cytoplasm [81], and in the response to TNF-α stimulation, Tβ4 was translocated into the nucleus with p65, and inhibited p65 from binding to the promoter of interleukin [82], thus exert anti-inflammatory effect via targeting NF-κB p65. Few studies reported Tβ4 exerts anti-inflammatory effect via targeting NF-κB p52, or any reports targeting Tβ4 to attenuate NF-κB signaling pathway and neuroinflammation in AD animal models. We thus design whether the effect of pathway intervention will be further improved based on Tβ4 intervention than using Tβ4 alone. Even though the inflammatory pathway proteins occurred to a lesser extent in combined TLR4 or NF-κB inhibition group, the index of phenotypic polarization of glial cells and neuron function, as well as the behavior performance, did not show a further improvement compared to Tβ4 intervention alone. So, there are solid grounds to believe that the signaling pathway responsible for these improvements was not restricted to anti-inflammatory signaling, we propose that a second major function of Tβ4 is to directly improve neuronal functions, which may open a new window of Tβ4 signaling. It is known that glial cells and neurons lie close together, and there is significant cross-talk between the TLR4/NF-κB and TNF-R/caspase signaling [83], and this may be the major site for simultaneously regulating neuron functions by neuroinflammation operation in our experimental model. Thus, we cannot rule out aspects of the direct neuromodulation following Tβ4 over-expression that neurons also over-expressed the Tβ4. The glia and neurons may act in tandem to promote the functional shift. It was necessary to clarify whether the effect was a direct or indirect effect. This study provides clues to explore the further precise mechanisms.

Concerning the limitations of this study, first is the fact that the limited number of mice used for behavior test, we could not assess whether there were sex differences in Tβ4 intervention mice. But Tβ4 gene escapes X inactivation and has a homolog on chromosome Y. Another limitation is the specific cellular targets of our lentiviral Tβ4 intervention cannot be firmly established from this study, and results therefore remain partly speculative. Similarly, we should use adult cell separation or fluorescence co-localization to more precisely determine the type of neuronal cell generating Aβ degrading enzyme. While in normal conditions Tβ4 is present inside the cells, it is secreted upon their stimulation and also has extracellular functions. And whether Tβ4 effects are mediated intracellularly, extracellularly or both, further studies will need to be done to confirm this hypothesis [18]. Nevertheless, the novelty of our work stems from the fact that our data give clues as to displaying a phenotype shift profile and a preliminary search of Tβ4 treatment for AD.

## Conclusion

**Fig. 12:**
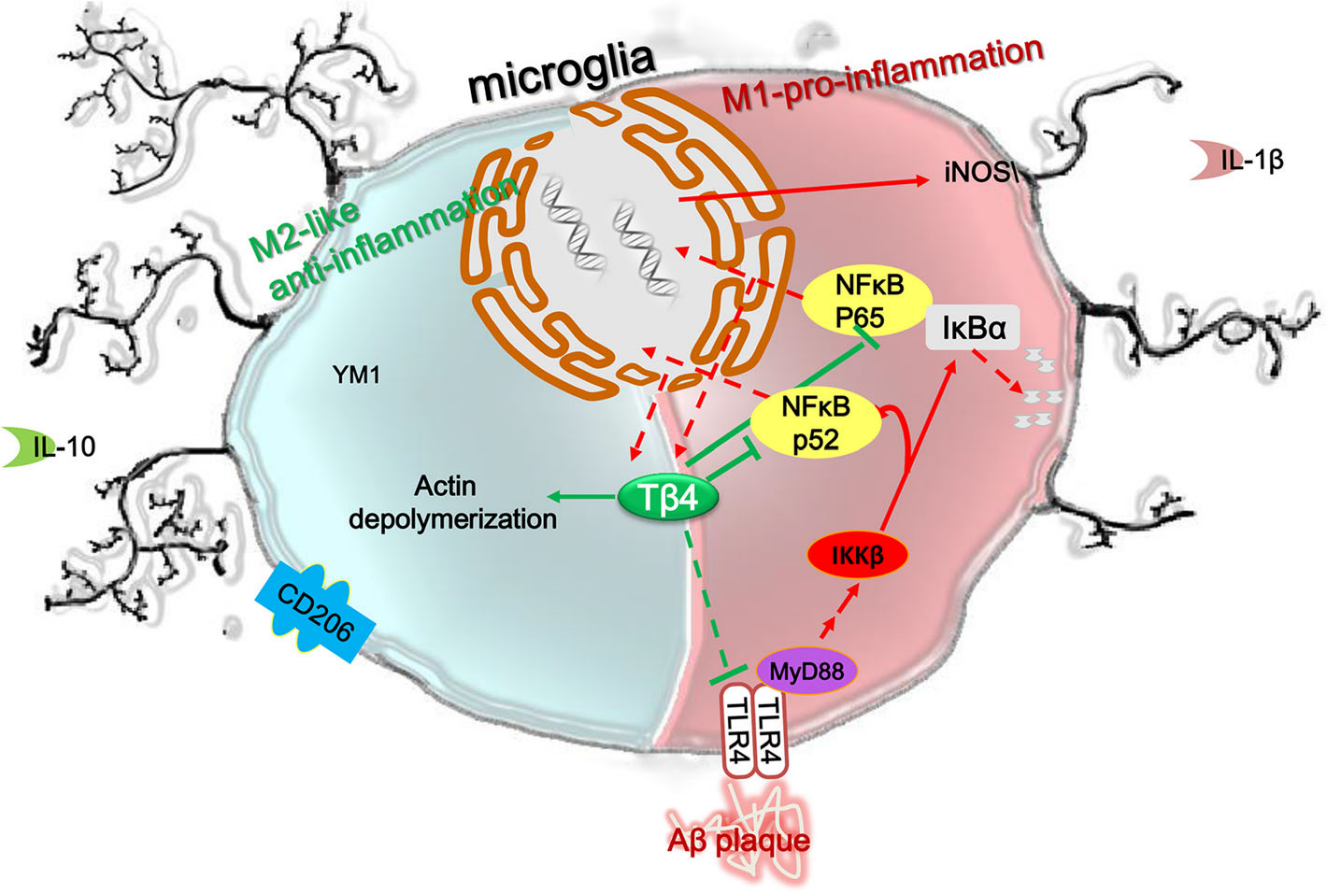
Diagram shows that Tβ4 promotes microglia phenotype shift from M1 to M2-like, and reduces production of pro-inflammatory cytokines or Aβ accumulation, improves cognitive decline, and displays antidepressant-like effect. Tβ4 intervention exerts obvious anti-inflammatory and neuroprotective effects in large part but not only by negative regulating TLR4/NF-κBp65 and NF-κBp52 signaling pathways in APP/PS1 mice

Overall, as shown in Fig. 12, we concluded that the Tβ4 intervention alone can exert obvious anti-inflammatory and neuroprotective effect in large part but not only by negative regulation of NF-κB pathway in APP/PS1 mice. The present study strengthen the ability of Tβ4 to target many diverse restorative processes via multiple molecular pathways that suppress inflammation, drive oligodendrogenesis and neurovascular remodeling, open avenues for new therapeutic applications to a range of neurodegenerative conditions [84]. Even though assessing the underlying mechanism manipulations is complicated, we have endeavored to acknowledge such inherent complexities when we feel they are critical for a proper comprehension of the relevant phenomenon, which is of great significance for its basic research and represent an important step in developing Tβ4 into an effective, tolerable, and safe pharmaceutical agent available to prevent, slow, halt, or reverse AD.

## Supplementary Information

**Additional file 1.**

**Additional file 2.**

## Data Availability

Not applicable.

## Abbreviations

5-HT_1A_R: 5-hydroxytryptamine 1A receptor
AD: Alzheimer’s disease
Aβ: Amyloid beta
ANOVA: Analysis of variance
AOD: Average optical density
BDNF: Brain-derived neurotrophic factor
CREB: cAMP-response element binding protein
DAB: Diaminobenzidine
DAPI: 4′,6-diamidino-2-phenylindole
DI: Discrimination index
DMSO: Dimethylsulfoxide
EAE: Experimental autoimmune-encephalomyelitis
ECL: Enhanced chemiluminescence
EDTA: Ethylene diamine tetraacetic acid
ELISA: Enzyme-linked immunosorbent assay
EPM: Elevated plus maze
Fizz1: Found in inflammatory zone 1/resistin-like-α
FST: Forced swimming test
GFAP: Glial fibrillary acidic protein
GluR1: Glutamate receptor 1
HEPES: 2-[4-(2-hydroxyethyl)piperazin-1-yl]ethanesulfonic aci
HRP: Horseradish peroxidase
Iba-1: Ionized calcium-binding adapter molecule 1
IDE: Insulin-degrading enzyme
IF: Immunofluorescence
IHC: Immunohistochemistry
IL-1β: Interleukin-1β
IL-10: Interleukin-10
iNOS: Inducible nitric oxide synthase
LV: Lentivirus
MAP 2: Microtubule-associated protein 2
MME: Membrane-metalloendopeptidase, also named neprilysin
MS: Multiple sclerosis
MWM: Morris water maze
MyD88: Myeloid differentiation factor 88
NeuN: Neuronal nuclei
NF-κB: Nuclear factor-κB
NORT: Novel object recognition test
TNF-α: Tumor necrosis factor-α
OCT: Optimum cutting temperature
OFT: Open field test
PBS: Phosphate buffer saline
PDE2: Phosphodiesterases
PDTC: Pyrrolidinedithiocarbamate
PFA: Paraformaldehyde
PSD95: Postsynaptic density-95kDa
qRT-PCR: Real-time quantitative reverse-transcription polymerase chain reaction
Tβ4: Thymosin beta4
TGF-β: Transforming growth factor-β
TLR4: Toll-like receptor 4
TNFR2: Tumor necrosis factor receptor
TRAF-6: Tumor necrosis factor receptor-associated factor 6
WT: Wild-type
YM1: Chitinase 3-like 3
