# Supplementary figures and images for: Thymosin β4 reverses phenotypic polarization of glial cells and cognitive impairment via negative regulation of NF-κB signaling axis in APP/PS1 mice
Source: J Neuroinflammation. 2021 Jun 28;18:146. doi: 10.1186/s12974-021-02166-3 (PMC8240373; doi:10.1186/s12974-021-02166-3)

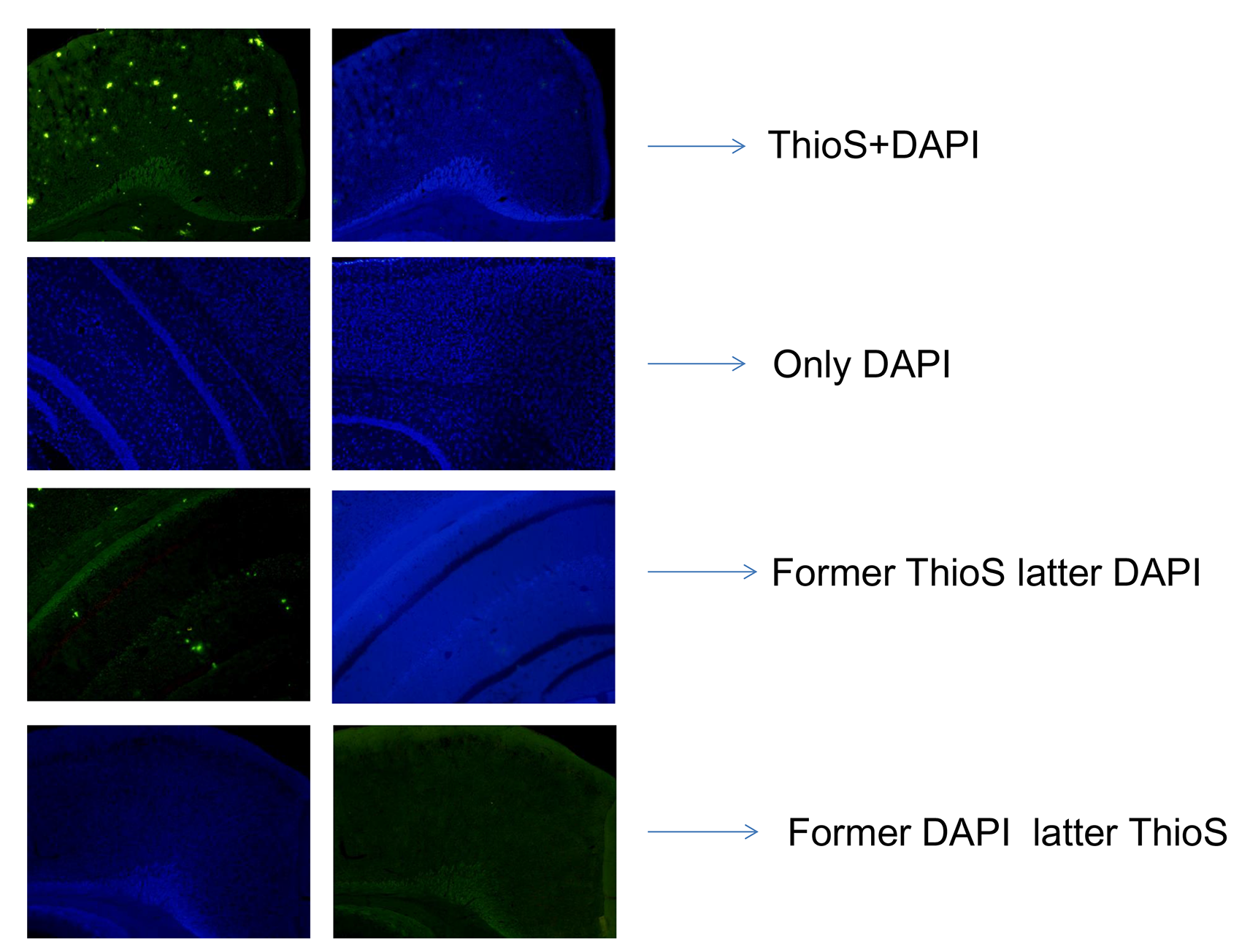

Supplement: Supplementary file 1 — Additional file 1. [file 12974_2021_2166_MOESM1_ESM.tif]

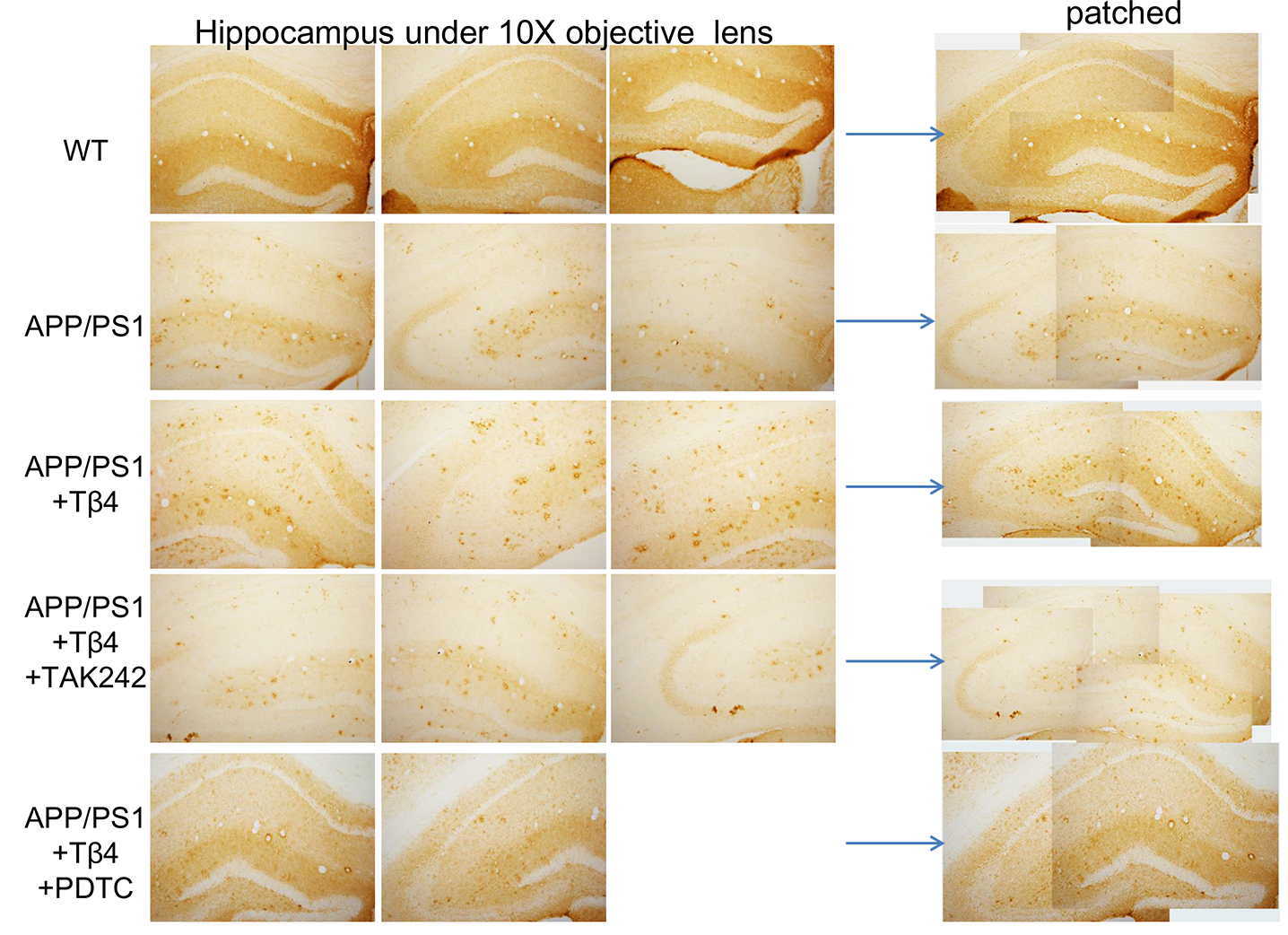

Supplement: Supplementary file 2 — Additional file 2. [file 12974_2021_2166_MOESM2_ESM.tif]
